# Automated orthogonal tRNA generation

**DOI:** 10.1038/s41589-024-01782-3

**Published:** 2024-12-20

**Authors:** Martin Spinck, Amir Guppy, Jason W. Chin

**Affiliations:** Medical Research Council Laboratory of Molecular Biology, Cambridge, UK

**Keywords:** Synthetic biology, High-throughput screening, RNA, Protein design

## Abstract

The ability to generate orthogonal, active tRNAs—central to genetic code expansion and reprogramming—is still fundamentally limited. In this study, we developed Chi-T, a method for the de novo generation of orthogonal tRNAs. Chi-T segments millions of isoacceptor tRNA sequences into parts and then assembles chimeric tRNAs from these parts. Chi-T fixes the parts, containing identity elements, and combinatorially varies all other parts to generate chimeric sequences. Chi-T also filters the variable parts and chimeric sequences to minimize host identity elements. We show here that experimentally characterized orthogonal tRNAs are more likely to have predicted minimum free energy cloverleaf structures, and Chi-T filters for sequences with a predicted cloverleaf structure. We report RS-ID for the identification of synthetases that may acylate the tRNAs generated by Chi-T. We computationally identified new orthogonal tRNAs and engineered an orthogonal pair generated by Chi-T/RS-ID to direct non-canonical amino acid incorporation, in response to both amber codons and sense codons, with an efficiency similar to benchmark genetic code expansion systems.

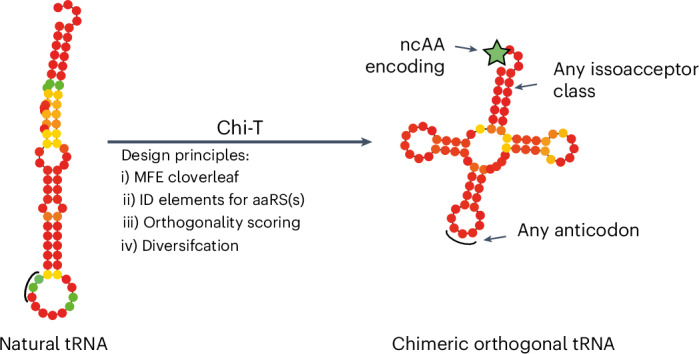

## Abstract (web summary)

Genetic code expansion and reprogramming require orthogonal tRNAs. Methods have now been developed for the automated generation of chimeric orthogonal tRNAs and discovery of their cognate synthetases. These approaches have been used to discover new orthogonal pairs for efficient non-canonical amino acid incorporation.

## Main

The discovery of orthogonal transfer RNAs (tRNAs) and their cognate aminoacyl-tRNA synthetases provides a foundation for genetic code expansion and genetic code reprogramming^1–4^. Orthogonal tRNAs must be transcribed and correctly processed in the host cell and not appreciably aminoacylated by endogenous aminoacyl-tRNA synthetases. To be useful for genetic code reprogramming, orthogonal tRNAs must also be directed to an otherwise unassigned codon, commonly through anticodon mutation, and fold into the correct three-dimensional structure to enable aminoacylation by their cognate aminoacyl-tRNA synthetase.

Orthogonal tRNAs have commonly been discovered by ad hoc processes^5–16^. We previously performed a systematic two-step computational and experimental search of 2.8 million tRNA sequences, from diverse bacteria, archaea, chloroplasts and bacteriophage, for tRNAs that are orthogonal in *Escherichia coli*^17^. We experimentally characterized 231 of the resulting tRNAs, for which we identified the corresponding synthetase gene. These experiments defined tRNA genes (with their native anticodon sequences) for which no transcript was detected (defined as undetectable) and tRNA genes with a detectable transcript that was (1) aminoacylated by *E. coli* synthetases (defined as non-orthogonal); (2) not aminoacylated in *E. coli* containing the cognate synthetase gene (defined as orthogonal inactive); and (3) not aminoacylated by *E. coli* synthetases but was aminoacylated by a co-expressed cognate synthetase (defined as orthogonal active).

The properties of several orthogonal active tRNAs were altered by anticodon mutation. For example, the orthogonal active tRNAs in *Sorangium cellulosum (Sc)* AspRS/*Sc*^Asp^tRNA_GUC_, *Ilumatobacter nonamiensis (In)* GlnRS/*In*^Gln^tRNA_UUG_ and *Coprobacillus sp. D7* (*Cs*) ProRS/*Cs*^Pro^tRNA_UGG_ pairs were converted to orthogonal inactive tRNAs upon mutating their anti-codon to CUA^17^. In some cases (for example, *Sc*^Asp^tRNA_CUA_ or *In*^Gln^tRNA_CUA_), we could convert these orthogonal inactive tRNA_CUA_s into orthogonal active tRNAs by evolving the anticodon recognition of the cognate synthetase. However, in other cases (for example, *Cs*^Pro^tRNA_CUA_), we could not re-activate the orthogonal inactive tRNAs by directed evolution. Orthogonal active tRNAs were also converted to non-orthogonal tRNAs upon mutating their anticodon to CUA. In some cases (for example, *Af*^Tyr^tRNA_CUA_), we could convert these non-orthogonal tRNAs into orthogonal active tRNAs, by directed evolution of the tRNA, followed by directed evolution of the anticodon recognition of the cognate synthetase. However, in other cases (for example, *Ap*^His^tRNA_CUA_), we did not regenerate orthogonal active tRNAs^17^.

Here we show that tRNAs, which are orthogonal and active in *E. coli*, are more likely to fold into a predicted minimum free energy (MFE) structure that is cloverleaf than tRNAs that are non-orthogonal or undetectable in *E. coli*. We leveraged our insights into the properties of orthogonal tRNAs to create orthogonal active tRNAs from both an inactive orthogonal tRNA (*Cs*^Pro^tRNA_CUA_) and a non-orthogonal tRNA (*Ap*^His^tRNA_CUA_). We developed Chi-T, a computational tool for automatically generating chimeric orthogonal tRNAs. Chi-T generates millions of chimeric isoacceptor tRNA sequences and then filters these chimeric sequences to directly identify a small number of diverse sequences that have minimal identity elements for *E. coli* synthetases and are predicted to fold into a robust cloverleaf. We also developed RS-ID to identify synthetases that may acylate the orthogonal tRNAs discovered through Chi-T. Using this approach, we directly discovered orthogonal tRNAs: 1092^Trp^tRNA_CUA_, which is active with *Pyrococcus horikoshii* TrpRS, and 1081^Arg^tRNA_CUA_, which is active with *Capnocytophaga sp*. ArgRS. Overall, we generated four new active and orthogonal tRNAs (*Cs*^Pro^tRNA_CUA_fix, T2H9^His^tRNA_CUA_fix, 1081^Arg^tRNA_CUA_ and 1092^Trp^tRNA_CUA_) with altered anticodons that redirect them to the amber stop codon. We evolved the anticodon recognition of the cognate synthetase for three of these pairs to create pairs that function with an efficiency similar to benchmark genetic code expansion systems. We further engineered the *Ph*TrpRS/1092^Trp^tRNA_CUA_ orthogonal pair for non-canonical amino acid (ncAA) incorporation.

## Results

### Predicted cloverleaf a hallmark of active and orthogonal tRNAs

**Fig. 1:**
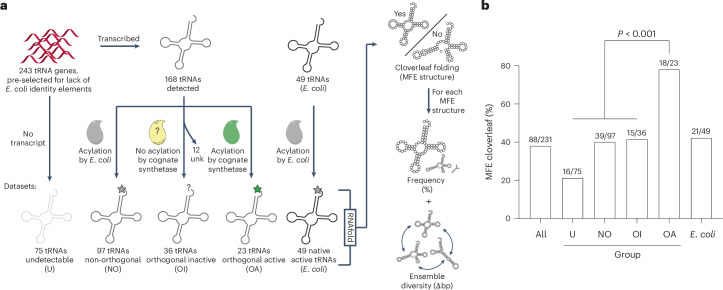
Active and orthogonal tRNAs commonly form predicted cloverleaf MFE structures. **a**, Classification of 243 tRNAs into four groups on the basis of previous tREX data^17^ that identified whether a tRNA was present in *E. coli* and whether it was acylated by endogenous synthetases or in the presence of the gene for its cognate synthetase (NO, non-orthogonal; OA, orthogonal active; OI, orthogonal inactive; U, undetectable). Orthogonal tRNAs for which the cognate synthase could not be identified (unk) were excluded from further analysis. For each group, the predicted MFE structure was calculated using RNAfold. We determined if the predicted structure matched an expected cloverleaf fold of a particular isoacceptor class (yes/no) by inspection. Minimum free energy structure frequency (percent contribution to the ensemble) and diversity (base pair distance between possible structures within the ensemble) were determined for all tRNAs (Supplementary Fig. 1). **b**, Percentage of sequences within each group that are predicted to form MFE cloverleaf structures; the corresponding percentage for *E. coli* tRNAs is also shown. The number of MFE cloverleaf tRNAs and total members of each group are shown. The observed distribution in the OA group diverges significantly (*P* *=* 5.3 × 10^−5^) from the expected distribution found for all other tested tRNAs. Statistical testing was performed using the two-tailed Fisher’s exact test. Source data

To investigate the relationship between tRNA structure, expression and activity, we calculated (using RNAfold^18^) the predicted MFE structure for each of the 231 tRNA sequences that we previously investigated^17^ and a reference set of 49 native *E. coli* tRNAs. We then checked whether the structure predicted by RNAfold matched the cloverleaf structure for a tRNA of the corresponding isoacceptor class. We also extracted the frequency—the percentage of the MFE structure in the predicted ensemble—and the diversity—the average base pair distance between possible alternative structures in the ensemble (Fig. 1a and Supplementary Fig. 1).

Approximately 40% of the primary sequences of native *E. coli* tRNAs were predicted to fold into cloverleaf tRNAs, providing a benchmark for the percentage of tRNA sequences predicted to fold into cloverleaf structures in the absence of host-specific factors. Thirty-eight percent of the 231 tRNAs in our dataset were also predicted to fold into cloverleaf tRNAs.

Among primary sequences of non-orthogonal active tRNAs—which are sufficiently similar to *E. coli* tRNAs to be aminoaclyated by endogenous synthetases—40.2% were predicted to fold into cloverleaf tRNAs; these tRNAs may be sufficiently similar to *E. coli* tRNAs that they benefit from mechanisms that help fold *E. coli* tRNAs.

Strikingly, approximately 80% of orthogonal active tRNAs are predicted to fold into a cloverleaf structure (Fig. 1b); this is a significantly higher percentage than expected (*P* < 0.001) and suggests that predicted MFE cloverleaf folding is a distinct feature of active and orthogonal tRNAs (Supplementary Note 1). These tRNAs may need to have the ability to fold into a cloverleaf hardwired into their primary sequence, as they may be sufficiently distinct from *E. coli* tRNAs that they do not benefit from mechanisms that help fold endogenous tRNAs^19,20^.

### Converting orthogonal tRNAs from inactive to active

Our analysis prompted us to investigate the effect of introducing mutations—that are predicted to increase the percentage of cloverleaf structures and decrease the diversity of other structures—for two tRNAs: *Cs*^Pro^tRNA_CUA_ and *Ap*^His^tRNA_CUA_. The parent tRNAs (*Cs*^Pro^tRNA_UGG_ and *Ap*^His^tRNA_GUG_) are orthogonal with respect to *E. coli* synthetases and are aminoacylated by their cognate synthetases (*Coprobacillus sp. D7* ProRS and *Afifella pfennigii DSM 17143* HisRS) and are, therefore, classed as orthogonal active tRNAs. However, *Cs*^Pro^tRNA_CUA_ does not direct stop codon read-through in *E. coli* in the presence of the cognate synthetase of its parent tRNA (and we class it as orthogonal inactive), whereas *Ap*^His^tRNA_CUA_ directs stop codon read-through in the absence of the cognate synthetase of its parent tRNA (and we class it as non-orthogonal active).

**Fig. 2:**
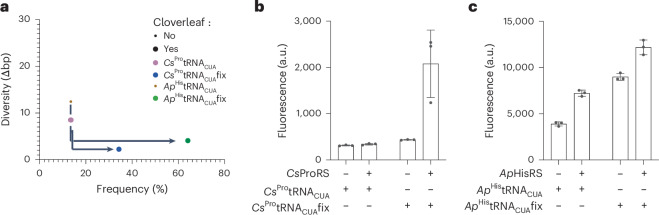
Fixing the predicted cloverleaf structure of an orthogonal inactive tRNA generates an orthogonal active tRNA, but fixing the cloverleaf structure of a non-orthogonal active tRNA is not sufficient to generate an orthogonal active tRNA. **a**, Frequency–diversity plots for the indicated tRNAs. *Cs*^Pro^tRNA_CUA_ and *Ap*^His^tRNA_CUA_, which exhibit weak or no cloverleaf folding, are connected to their structurally fixed derivatives by arrows (Supplementary Fig. 2). Cloverleaf MFE structures are indicated with large dots. Non-cloverleaf MFE structures are indicated with small dots. **b**, Fixing the predicted cloverleaf structure of *Cs*^Pro^tRNA_CUA_ (an orthogonal inactive tRNA) generates an orthogonal active tRNA, *Cs*^Pro^tRNA_CUA_fix. GFP fluorescence was measured in cells containing *sfGFP3TAG*_*His6*_ and *Cs*^Pro^tRNA_CUA_ or *Cs*^Pro^tRNA_CUA_fix with or without *Cs*ProRS. GFP fluorescence generated from *sfGFP3TAG*_*His6*_ by the *Mm*PylRS/*Mm*^Pyl^tRNA_CUA_ with 2 mM AllocK under the same conditions was approximately 20,000 a.u. The experiments were performed in three independent replicates. The individual data points are shown as dots; the bars represent mean values; and the error bar indicates the standard deviation. **c**, Fixing the predicted cloverleaf structure of *Ap*^His^tRNA_CUA_ (a non-orthogonal active tRNA) generates a more active non-orthogonal tRNA, *Ap*^His^tRNA_CUA_fix. GFP fluorescence was measured in cells containing *sfGFP3TAG*_*His6*_ and *Ap*^His^tRNA_CUA_ or *Ap*^His^tRNA_CUA_fix with or without *Ap*HisRS. GFP fluorescence generated from *sfGFP3TAG*_*His6*_ by the *Mm*PylRS/*Mm*^Pyl^tRNA_CUA_ with 2 mM AllocK under the same conditions was approximately 20,000 a.u. The experiments were performed in three independent replicates. The individual data points are shown as dots; the bars represent mean values; and the error bar indicates the standard deviation. Source data

*Cs*^Pro^tRNA_CUA_ has a low percentage predicted cloverleaf structure in the ensemble, and *Ap*^His^tRNA_CUA_ does not fold into an unambiguous predicted cloverleaf structure (Supplementary Fig. 2). We fixed both tRNA structures into high-frequency/low-diversity predicted cloverleaf structures with 2–3 rationally designed point mutations (Fig. 2 and Supplementary Fig. 2). We expressed the derivatives, *Cs*^Pro^tRNA_CUA_fix and *Ap*^His^tRNA_CUA_fix, and measured the ability of each tRNA (with and without the cognate synthetase of the parent tRNA) to read through an amber stop codon at position 3 of GFP, in *sfGFP3TAG*_*His6*_, to generate a fluorescent signal (Fig. 2b,c).

*Cs*^Pro^tRNA_CUA_fix exhibited a six-fold increase in activity over *Cs*^Pro^tRNA_CUA_ in the presence of *Cs*ProRS and had little activity in the absence of this synthetase. We note that this pair is much less active (10-fold) than the *Mm*PylRS/*Mm*^Pyl^tRNA_CUA_ pair currently used for genetic code expansion. The encoding of proline with the *Cs*ProRS/*Cs*^Pro^tRNA_CUA_fix pair was confirmed by mass spectrometry (Supplementary Fig. 3). We conclude that introducing mutations that fix the predicted cloverleaf structure of this tRNA is sufficient to convert it from an orthogonal inactive tRNA to an orthogonal active tRNA in *E. coli*; this is consistent with our hypothesis that *Cs*^Pro^tRNA_CUA_ is inactive due to mis-folding, but other interpretations are formally possible. *Ap*^His^tRNA_CUA_fix was further activated with respect to *Ap*^His^tRNA_CUA_ but remained non-orthogonal active. We conclude that fixing the predicted cloverleaf structure of a non-orthogonal active tRNA is not sufficient to generate an orthogonal active tRNA. Overall, we suggest that fixing the cloverleaf structure of a tRNA has the potential to increase its activity but does not necessarily increase its orthogonality.

### Converting non-orthogonal tRNAs to orthogonal tRNAs

**Fig. 3:**
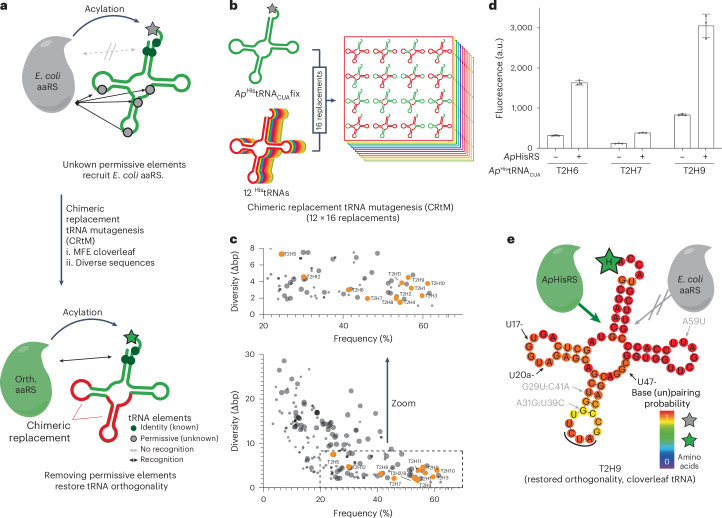
Generating an orthogonal active tRNA from a non-orthogonal active tRNA via CRtM. **a**, A heterologous tRNA of interest (green), with minimal *E. coli* identity elements, and identity elements for the cognate synthetase (green circles) in aminoacylated with a canonical amino acid (gray star) by *E. coli* aminoacyl-tRNA synthetases (gray). This acylation is a consequence of permissive tRNA elements (gray circles) and makes the tRNA non-orthogonal. We generated an orthogonal tRNA through CRtM. CRtM systematically replaces sections of a tRNA with sequences from other isoacceptor tRNAs with diverse sequences. This approach aims to remove permissive elements while maintaining an MFE cloverleaf structure. Some chimeric tRNAs no longer have permissive elements and maintain activity with the cognate orthogonal aminoacyl tRNA synthetase; these tRNAs are orthogonal and active. **b**, We computationally replaced defined regions of *Ap*^His^tRNA_CUA_fix sequence (green) with the corresponding regions from 12 histidine tRNAs with minimal *E. coli* identity elements (colored). These replacements followed 16 defined replacement schemes and generated 192 chimeric sequences. **c**, We used RNAfold to predict the frequency and diversity of all 192 chimeric tRNAs. Chimeras that fold into a cloverleaf are indicated by larger circles; any other folding is depicted as small circles. The diversity and frequency of the final set of 12 chimeric tRNAs (T2H1–TH12) selected for experimental validation are shown in orange (selection and optimization detailed in Supplementary Fig. 5 and Supplementary Tables 1–3). **d**, GFP fluorescence was measured in cells containing *sfGFP3TAG*_*His6*_ and the indicated *Ap*^His^tRNA_CUA_fix derivative, with or without *Ap*HisRS (other derivatives in Supplementary Fig. 4a). The experiments were performed in three independent replicates. The individual data points are shown as dots; the bars represent mean values; and the error bar indicates the standard deviation. **e**, MFE structure prediction for T2H9^His^tRNA_CUA_fix. Base (un)pairing probability is colored. Sequence changes with respect to *Ap*^His^tRNA_CUA_fix are indicated by arrows; gray arrows indicate changes unique to T2H9^His^tRNA_CUA_fix; black arrows indicate changes found for all orthogonal tRNAs (T2H9, T2H6 and T2H7). Encoding of histidine (green star) was confirmed for T2H6, T2H7 and T2H9 by mass spectrometry (Supplementary Fig. 4b). Source data

We demonstrated that *Ap*^His^tRNA_CUA_fix is aminoacylated with either lysine or glutamine (Supplementary Fig. 4), as found for *Ap*^His^tRNA_CUA_^17^. *Ap*^His^tRNA_CUA_ and *Ap*^His^tRNA_CUA_fix do not contain the set of classical identity elements for *E. coli* lysyl-tRNA or glutaminyl-tRNA synthetases. We, therefore, hypothesized that unknown sequence elements within these tRNAs were responsible for their aminoacylation by *E. coli* synthetases. As the location and nature of these permissive elements were unknown, we aimed to diversify the whole sequence of *Ap*^His^tRNA_CUA_fix by a strategy we refer to as Chimeric Replacement tRNA Mutagenesis (CRtM) (Fig. 3a). This approach aims to generate chimeric sequences that (1) retain a high-frequency MFE cloverleaf structure, (2) cover diverse sequences and (3) minimize the canonical identity elements for *E. coli* synthetases. We anticipated that CRtM may provide a pool of sequences enriched with active and orthogonal tRNAs.

We generated chimeras of *Ap*^His^tRNA_CUA_fix by substituting defined sections in the tRNA body, using 16 replacement schemes (Fig. 3b), with sequences from 12 selected histidyl-tRNAs; these tRNAs were chosen because they have minimal canonical *E. coli* identity element nucleotides (Supplementary Table 1)^17^. This created 192 chimeric tRNA sequences (16 × 12; Supplementary Table 2). We calculated the frequency of the most abundant predicted structure and the diversity of predicted structures for each sequence using RNAfold (Fig. 3c). We chose chimeric sequences to characterize (Supplementary Table 3) such that (1) each of the 12 selected tRNAs was represented; (2) the sequences had a predicted cloverleaf structure as their most abundant species; and (3) the predicted cloverleaf folding was unambiguous. For five of the 12 ^His^tRNAs, such a chimeric tRNA was directly generated by one of the replacement schemes. We further optimized the remaining seven chimeric tRNAs by manually introducing point mutations (Fig. 3c and Supplementary Fig. 5), which increased unambiguous predicted cloverleaf folding or removed potential identity element nucleotides for *E. coli* synthetases.

The 12 selected and optimized chimeric tRNAs were tested for their ability to mediate read-through of the amber stop codon in *sfGFP3TAG*_*His6*_ with and without *Ap*HisRS (Fig. 3d and Supplementary Fig. 4). Six of the ^His^tRNA_CUA_s (T2H1, T2H3, T2H4, T2H8, T2H11 and T2H12) exhibited little activity regardless of whether *Ap*HisRS was added, and three tRNAs (T2H2, T2H5 and T2H10) were active and exhibited little change in activity upon addition of *Ap*HisRS; these tRNAs are non-orthogonal and were not considered further (Supplementary Fig. 4a). Three tRNAs (T2H6, T2H7 and T2H9) exhibited *Ap*HisRS-dependent read-through of the amber codon (Fig. 3d); these tRNAs exhibited some orthogonality. tRNAs T2H6, T2H7 and T2H9—unlike *Ap*^His^tRNA_CUA_fix—directed the incorporation of histidine in response to the amber stop codon in *sfGFP3TAG*_*His6*_, when paired with *Ap*HisRS (Supplementary Fig. 4b).

Comparing the sequence of *Ap*^His^tRNA_CUA_fix, which is aminoacylated by *E. coli* synthetases with lysine or glutamine, and the T2H6, T2H7 and T2H9 chimeras, which are selectively aminoacylated by *Ap*HisRS with histidine, reveals that all the chimeric tRNAs share 2-base deletions in the D-loop and have a 1-nucleotide (nt) shorter variable loop (Fig. 3e). The deleted bases of the D-loop are not canonical identity elements for the *E. coli* aminoacyl-tRNA synthetases that direct the aminoacylation of their cognate tRNAs with lysine or glutamine^21^. Instead, the deleted bases are likely ‘permissive elements’ that permit the mis-aminoacylation of these tRNAs by particular *E. coli* synthetases^22,23^. The deletions—within the context of these chimeric tRNAs—may be anti-determinants for mis-aminoacylation by these *E. coli* synthetases^21^.

Our data demonstrate how permissive elements for the mis-aminoacylation of a tRNA, which are much more poorly understood and delineated than identity elements, can be removed through the targeted exploration of tRNA sequence diversity.

### Evolved anti-codon recognition enhances pair activity

**Fig. 4:**
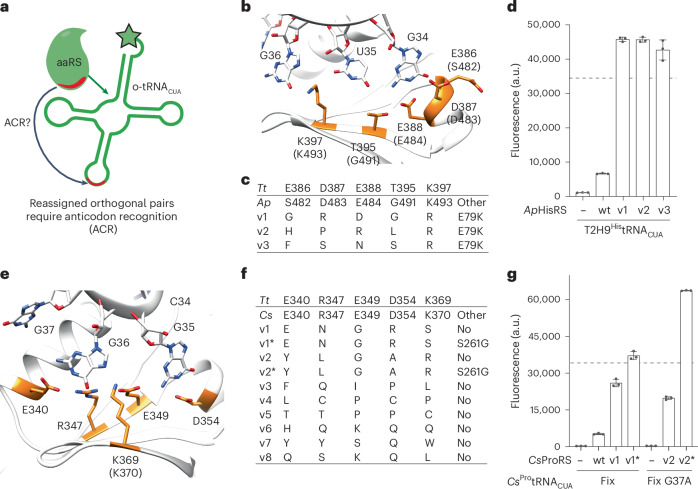
Directed evolution of synthetase ACR generates synthetases that function with orthogonal tRNA with similar activity to benchmark genetic code expansion systems. **a**, T2H9^His^tRNA_CUA_fix and *Cs*^Pro^tRNA_CUA_fix contain altered anticodons that may not be efficiently recognized by *Ap*HisRS and *Cs*ProRS, respectively. **b**, Anticodon recognition in the *T. thermophilus (Tt)HisRS*/^His^tRNA_GUG_ pair (PDB: 4RDX). Anticodon bases are shown in white. Residues in *Tt*HisRS that recognize the anticodon are shown in orange; the numbering for the corresponding residues in *Ap*HisRS is shown in brackets—these residues were targeted for mutagenesis in the ACR library of *Ap*HisRS. **c**, Sequences of *Ap*HisRS variants obtained from the ACR library after a single round of selection (Supplementary Fig. 6). **d**, GFP fluorescence was measured in cells containing T2H9^His^tRNA_CUA_fix and a *sfGFP3TAG*_*His6*_ gene. Cells contained the indicated *Ap*HisRS variant: no synthetase (−), *Ap*HisRS (wt) or its evolved variants (v1–3). The dashed line indicates the level of GFP fluorescence generated from *sfGFP3TAG*_*His6*_ by the *Mm*PylRS/*Mm*^Pyl^tRNA_CUA_ with 2 mM AllocK. The experiments were performed in three independent replicates. The individual data points are shown as dots; the bars represent mean values; and the error bars indicate the standard deviation. **e**, ACR in the *Tt*ProRS/*Tt*^Pro^tRNA_UGG_ pair (PDB: 1HQ4). Anticodon bases and base G37 are shown in white. Residues in *Tt*ProRS that recognize the anticodon are shown in orange; the numbering for the corresponding residues in *Cs*ProRS, where different, is shown in brackets—these residues were targeted for mutagenesis in the ACR library of *Cs*ProRS. **f**, Sequences of *Cs*ProRS variants obtained from the ACR library after a single round of selection (Supplementary Fig. 7). The *Cs*ProRS v2 and v3 sequences were isolated with *Cs*^Pro^tRNA_CUA_fix (G37A), a spontaneous mutant of the tRNA used as an input for the selection (Supplementary Table 6). We also generated variants of the selected *Cs*ProRS mutants, indicated with an *; we transplanted the S261G mutation into these variants (*Ec*ProRS C443G)^26^. **g**, GFP fluorescence from *sfGFP3TAG*_*His6*_ measured in cells containing *Cs*^Pro^tRNA_CUA_fix or *Cs*^Pro^tRNA_CUA_fix G37A and the indicated *Cs*ProRS variant: no synthetase (−), *Cs*ProRS (wt) or its evolved variants (v1 and v2). The dashed line indicates the level of GFP fluorescence generated from *sfGFP3TAG*_*His6*_ by the *Mm*PylRS/*Mm*^Pyl^tRNA_CUA_ with 2 mM AllocK. The experiments were performed in three independent replicates. The individual data points are shown as dots; the bars represent mean values; and the error bars indicate the standard deviation. PDB, Protein Data Bank; wt, wild-type. Source data

The *Ap*HisRS/T2H9^His^tRNA_CUA_fix pair and the *Cs*ProRS/*Cs*^Pro^tRNA_CUA_fix pair that we developed exhibited 5–10% of the activity of the *Mm*PylRS/*Mm*^Pyl^tRNA_CUA_ pair commonly used for genetic code expansion. Because the anticodon of their cognate tRNAs is a recognition element for HisRS^24^ and ProRS^25^, we hypothesized that the activity of these pairs may be limited by inefficient aminoacylation of their tRNAs. Therefore, we investigated the directed evolution of *Ap*HisRS and *Cs*ProRS for improved activity with T2H9^His^tRNA_CUA_fix and *Cs*^Pro^tRNA_CUA_fix, respectively (Fig. 4a).

The structure of *Thermus thermophilus Tt*HisRS/*Tt*^His^tRNA_GUG_ reveals how the synthetase recognizes the anticodon of its cognate tRNA^24^ (Fig. 4b). We created an anticodon recognition (ACR) library in *Ap*HisRS by mutating residues Ser482, Asp483, Glu484 (responsible for recognition of G34 in the tRNA), Gly491 (proximal to G34) and Arg493 (which interacts with G36). All five positions were mutated to the 20 canonical amino acids, creating a library of 10^7^ mutants. The ACR library was subjected to selection for the ability to read through an amber stop codon at position 111 of chloramphenicol acetyltransferase (*CAT111TAG*) and confer resistance to chloramphenicol, when provided with T2H9^His^tRNA_CUA_fix (Supplementary Fig. 6). After selection, we isolated three distinct *Ap*HisRS variants (v1, v2 and v3; Fig. 4c). *Ap*HisRS(v1–3)/T2H9^His^tRNA_CUA_fix pairs were approximately seven times more active than the *Ap*HisRS/T2H9^His^tRNA_CUA_fix pair, when measured by read-through of an amber stop codon at position 3 of sfGFP (*sfGFP3TAG*; Fig. 4d).

The structure of *Tt*ProRS/*Tt*tRNA_CGG_ reveals how the synthetase recognizes the anticodon of its cognate tRNA^25^ (Fig. 4e). We created an ACR library by targeting residues Glu340, Arg347, Lys370, Glu349 and Asp354 in *Cs*ProRS. All five positions were mutated to the 20 canonical amino acids, creating a library of 10^7^ mutants. We subjected the ACR library to selection for the ability to read through the amber stop codon in *CAT111TAG* and confer chloramphenicol resistance, when provided with *Cs*^Pro^tRNA_CUA_fix. From the selection and subsequent screening (Supplementary Fig. 7), we identified eight *Cs*ProRS variants (v1–8; Fig. 4f).

The *Cs*ProRSv1/*Cs*^Pro^tRNA_CUA_fix pair showed a five-fold increase in activity with respect to the *Cs*ProRS/*Cs*^Pro^tRNA_CUA_fix pair. We introduced an S261G mutation (homologous to the C443G mutation previously introduced into *Ec*ProRS^26^) into *Cs*ProRSv1, creating *Cs*ProRSv1*. The *Cs*ProRSv1*/Cs^Pro^tRNA_CUA_fix pair was 1.5 times more active than the *Cs*ProRSv1*/Cs*^Pro^tRNA_CUA_fix pair.

During the evolution of *Cs*ProRSv2 and *Cs*ProRSv3, *Cs*^Pro^tRNA_CUA_fix spontaneously acquired a G37A mutation. This mutation is known to improve amber decoding in other tRNAs, including *Archaeoglobus fulgidus* ^Pro^tRNA_CUA_^7^. We confirmed that *Cs*^Pro^tRNA_CUA_fix (G37A) was orthogonal with respect to *E. coli* aminoacyl synthetases (Fig. 4g). The *Cs*ProRSv2/*Cs*^Pro^tRNA_CUA_fix (G37A) pair showed a four-fold increase in activity with respect to the *Cs*ProRS/*Cs*^Pro^tRNA_CUA_fix pair. We introduced the S261G mutation (homologous to the C443G mutation previously introduced into *Ec*ProRS^26^) into *Cs*ProRSv2, creating *Cs*ProRSv2*. The resulting *Cs*ProRSv2*/*Cs*^Pro^tRNA_CUA_fix (G37A) pair was 12 times more active than the *Cs*ProRS/*Cs*^Pro^tRNA_CUA_fix pair.

The activities of the *Ap*HisRS(v1–3)/T2H9^His^tRNA_CUA_fix and *Cs*ProRSv2*/*Cs*^Pro^tRNA_CUA_fix (G37A) pairs are similar to the activity of the *Mm*PylRS/^Pyl^tRNA_CUA_ pair commonly used for genetic code expansion. We conclude that orthogonal tRNAs and their cognate synthetase can be generated by (1) creating tRNAs that retain the identity elements for their cognate aminoacyl-tRNA synthetases and fold into a cloverleaf; (2) removing host (*E. coli*) identity elements and permissive elements to create tRNAs that are not aminoacylated by endogenous synthetases; and then (3) evolving the ACR of the cognate synthetase to ensure efficient aminoacylation.

### Chi-T and RS-ID generate orthogonal pairs

**Fig. 5:**
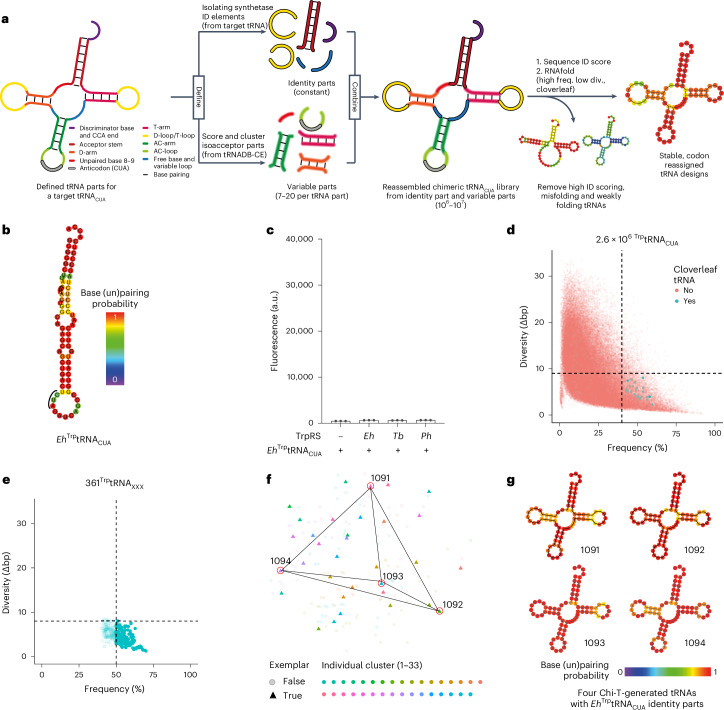
Chi-T, a computational algorithm for the de novo design of codon-reassigned, orthogonal tRNAs. **a**, The Chi-T workflow. **b**, RNAfold-predicted MFE structure of the *E. histolytica* tryptophanyl tRNA with its anticodon (black line) changed from CCG to CUA. Base coloring represents the predicted probability that a base has the pairing status predicted by the MFE structure. **c**, GFP fluorescence from *sfGFP3TAG*_*His6*_ measured in cells containing *E. histolytica* (*Eh*)^Trp^tRNA_CUA_ with no synthetase (−) or the tryptophanyl synthetases from *E. histolytica*, *T. brucei* and *P. horikoshii*. The experiments were performed in three independent replicates. The individual data points are shown as dots; the bars represent mean values; and the error bars indicate the standard deviation. **d**, Selection of ^Trp^tRNA_CUA_s with fixed *Eh*^Trp^tRNA identity parts and minimal *E. coli* identity elements that form cloverleaf structures. Sequences for which the MFE structure is a cloverleaf are shown as blue dots; all other sequences are shown as red dots. In total, 642 tRNAs were selected (solid blue dots); these had a frequency of cloverleaf greater than or equal to 40% and a diversity (Δbp) of 9 bp or less. All other sequences are represented by translucent dots. For simplicity of rendering, the graph shows data for 10^5^ randomly chosen chimeras. **e**, Selection of ^Trp^tRNA_CUA_s with fixed *Eh*^Trp^tRNA identity parts that are robust to anticodon mutation. The anticodons of the tRNAs from the selection step shown in **d** were varied to CUA, UGA and CGA. The 169 chimeric tRNA sequences passing these filters for this step are shown as blue dots in the frequency–diversity plot (Supplementary Fig. 13). **f**, Multidimensional scaling plot of the relationship among the final 169 *Eh*^Trp^tRNA chimeric sequences. Distances were computed as the Levenshtein distance between sequences, and sequences were clustered. Sequences are colored by cluster. Exemplar sequences, for each of the 33 clusters, are represented by solid triangles; all other sequences are represented by translucent dots. The four final tRNAs (1091, 1092, 1093 and 1094) are circled. Multidimensional scaling distances are not linearly correlated to the spatial distance on the plot. **g**, The RNAfold-predicted MFE structures of the indicated tRNAs (sequence in Supplementary Fig. 15). Source data

Next, we aimed to combine what we had learned about the properties of orthogonal tRNAs to create an approach for the computational generation of potentially active and orthogonal tRNAs from many more tRNA sequences. We started with the following postulates: (1) sequences that are predicted to fold into unambiguous cloverleaf structures will favor activity in *E. coli*; (2) recognition of a chimeric tRNA by a synthetase is favored when the identity elements of the cognate tRNA for the synthetase are present in the chimeric tRNA; (3) orthogonality of a cloverleaf tRNA will be determined by its sequence differences from *E. coli* tRNAs, particularly at identity element nucleotides; and (4) tRNA sequences that minimize permissive elements (which allow non-orthogonal interactions) and inhibitory elements (which abrogate activity) may be discovered by sampling diverse sequences from the same isoacceptor class. These postulates were incorporated into Chi-T, a computational algorithm that automatically generates a user-defined number of chimeric, unambiguous cloverleaf tRNA designs for a target anticodon or anticodons (Fig. 5a).

Chi-T takes the 10 million annotated and aligned tRNA sequences in tRNADB-CE^27^ as an input and automatically generates chimeric tRNA sequences. These sequences are assembled from nine distinct parts. These parts and their canonical tRNA numbering are as follows: acceptor stem {1–7 + 66–72}, unpaired bases 8–9 {8–9}, D-arm {10–13 + 22–25}, D/T-loops {14–21 + 54–60}, unpaired base and variable loop {26 + 44–48}, anticodon stem {27–31 + 39–43}, anticodon stem loop {32–38}, T-arm {49–53 + 61–65} and the discriminator base with CCA {73–76}. These parts are structurally defined, such that base pairing interactions and tertiary interactions are between nucleotide positions within a part.

Chi-T extracts the tRNA part sequences known to contain identity elements^21^ from a user-defined tRNA gene of interest and fixes these part sequences for all the chimeric tRNA sequences that it generates (Fig. 5a). Chi-T then varies the sequences of the remaining parts of the tRNA sequence, which do not contain known identity elements for the synthetases in the isoacceptor class of the user-defined tRNA. To choose the variable sequences for each part, Chi-T extracts all other tRNA sequences in the same isoacceptor class found in the tRNADB-CE database and sections these into parts, which are then filtered based on a number of user-defined criteria. The default settings for filtering are as follows: part sequences contain only nucleotides A, C, U and G (some database sequences contain N, R, etc.); sequences of paired parts (for example, D-arm) must be composed of Watson–Crick base pairs; variable loops must be shorter than 8 nt; unpaired bases 8–9 must contain at least one uracil nucleotide; and the D-loop must start with an adenine nucleotide.

Chi-T then compares each filtered part sequence to the corresponding part sequence of all *E. coli* tRNAs and, thereby, generates a part identity score; this score reflects the number of identity element nucleotides that each sequence part shares with the corresponding part sequences of all *E. coli* tRNAs (Methods and Supplementary Fig. 8). Part sequences with the lowest part identity scores contain the fewest *E. coli* identity elements. We, therefore, predicted that assembling chimeric tRNAs from part sequences with low part identity scores would favor the generation of tRNAs that are not aminoacylated by *E. coli* synthetases. For each part, Chi-T selects several part sequences (on the order of 10^1^–10^2^) with the lowest part identity scores. The selected part sequences are then clustered by affinity propagation, and Chi-T selects one exemplar part sequence from each distinct cluster with which to generate chimeric tRNAs. These steps aim to maximize both the dissimilarity of parts to their corresponding *E. coli* part sequences and the sequence diversity covered for each part.

Chi-T then combines the fixed and variable part sequences to form a library of chimeric tRNA sequences (Fig. 5a). The library is then optionally filtered by length (by default, tRNAs >78 nt are filtered out). The sequence identity score^17^ is determined for each chimera, and tRNA sequences with the highest sequence identity score (sharing the most identity elements with *E. coli* synthetases) are discarded. The stringency of filtering here is left to the user; however, Chi-T aims to keep 2.5 million tRNAs to balance low sequence identity scores, sufficient sequence diversity and computational memory and time efficiency.

The resulting library of chimeric tRNA sequences is then computationally folded, with RNAfold, to generate the MFE structure, frequency and ensemble diversity for each chimeric sequence. MFE structures that match the general stable cloverleaf structure, which we define within Chi-T but can be modified as a user input (Supplementary Fig. 9), are selected (the default cutoff points for diversity and cloverleaf frequency are set at diversity <10 Δbp and frequency >30%, but these can be varied within Chi-T). All tRNAs for which the predicted MFE structures are not cloverleaf, or for which the cloverleaf structure forms only a small part of the predicted conformational ensemble (low frequency), or for which the cloverleaf structure is not predicted to be stable (high diversity), are discarded.

Chi-T alters the anticodon of the tRNA sequences to each user-defined anticodon, in series, and repeats the structural prediction and filtering. The tRNA sequences with an average frequency and an average ensemble diversity above user-defined thresholds (across the defined anticodons) are taken forward. This kind of intrinsic robustness to anticodon reassignment is found in the *Mm*PylRS/*Mm*^Pyl^tRNA_CUA_ pair and its CGA and UGA anticodon variants: all three *Mm*^Pyl^tRNA anticodon variants are predicted by RNAfold to form robust cloverleaf structures (Supplementary Fig. 10), and the resulting pairs can decode their cognate codons^3^.

The resulting chimeric tRNA sequences are then clustered by affinity propagation to generate a set of tRNA sequences that represent the remaining sequence space. A user-defined number of output sequences is then identified for experimental characterization by calculating the group of tRNA sequences that maximize dissimilarity between any pair of output sequences.

To identify synthetases to test with the sequences generated by Chi-T, we developed RS-ID. This script takes all tRNA sequences from the same isoacceptor class used by Chi-T and identifies the subset of tRNAs with similar or identical identity element sequences and identity element parts to those of the tRNA used to define the fixed identity parts used in Chi-T. The organisms from which these tRNAs are derived provide candidate synthetases to test for activity with Chi-T-derived tRNAs (Supplementary Fig. 11). Testing several synthetases may increase the chance of finding a synthetase that expresses in a functional form in *E. coli* and that acylates the chimeras.

We tested Chi-T using the non-anticodon identity parts of ^Trp^tRNA from *Entamoeba histolytica* (the acceptor stem part (nucleotides 1–7 GGGGGCT and nucleotides 66–72 AGCCCTC) and the discriminator base with CCA part (nucleotides 73–76 ACCA)). These identity parts contain distinct nucleotides from *E. coli* ^Trp^tRNA at four of the seven, non-anticodon, identity element positions for Trp isoacceptors (nucleotides 1, 2, 3, 70, 71, 72 and 73) and do not contain a full set of identity element nucleotides recognized by any *E. coli* synthetase. RNAfold predicts a non-cloverleaf MFE structure for *Eh*^Trp^tRNA_CUA_ (Fig. 5b; frequency: 13.3%, diversity: 5.3 Δbp, cloverleaf: no). Expression of *Eh*^Trp^tRNA_CUA_ in *E. coli* does not lead to read-through of the amber stop codon in *sfGFP150TAG*_*His6*_. Combining *Eh*TrpRS, which can be expressed in *E. coli*^28^, with *Eh*^Trp^tRNA_CUA_ did not lead to enhanced GFP fluorescence from *sfGFP150TAG*_*His6*_ (Fig. 5c). These observations suggest that *Eh*^Trp^tRNA_CUA_ is not produced in a functional, stable form in *E. coli*.

To generate chimeric tRNAs with the identity elements from *Eh*^Trp^tRNA, Chi-T first extracted the two parts of *Eh*^Trp^tRNA that contain the non-anticodon identity elements for tryptophanyl-tRNA synthetases from tRNADB-CE and fixed these parts for all chimeric tRNAs. For the remaining seven parts, Chi-T extracted all unique sequences derived from tryptophanyl tRNAs from tRNADB-CE and created a library of variable part sequences for each part (Supplementary Fig. 12).

Chi-T scored all variable part sequences by their part identity score and selected the top 65 sequences for each part for clustering by affinity propagation (65 sequences were user defined to produce a computationally feasible number of chimeras from combining variable part sequences). Parts with fewer than 15 unique sequences, namely unpaired bases 8–9 with six unique sequences, were not clustered. Part sequences for all other parts were clustered individually. Cluster exemplars (D-arm: 8 part sequences, D/T-loop: 15 part sequences, unpaired base/variable loop: 14 part sequences, anticodon stem: 11 part sequences, anticodon stem loop: 9 part sequences, T-arm: 9 part sequences), along with non-clustered variable sequences (unpaired bases 8–9: 6 part sequences) and the fixed identity parts (acceptor stem and discriminator base with CCA), were used for chimera generation.

Combining the fixed and variable parts, and setting the anticodon to CUA, generated a library of 9.0 × 10^6^ (1 × 6 × 8 × 15 × 14 × 11 × 9 × 9 × 1) chimeric sequences, of which 7.5 × 10^6^ were shorter than 79 nt in length and were retained. In total, 2.6 × 10^6^ of these sequences passed the sequence identity score filter (sequences with a sequence identity score >0 for any isoacceptor class were discarded). We computationally folded the remaining sequences using RNAfold and discarded sequences whose MFE structures were not cloverleaf. Of the MFE cloverleaf tRNAs, we discarded those with a diversity >9 Δbp or cloverleaf structure frequency <40%. This step removed more than 99.9% of sequences, leaving 642 chimeric sequences that passed the first round of computational selection (Fig. 5d).

We decided to iteratively alter the anticodon of the chimeric tRNAs to UGA and CGA and remove tRNA sequences predicted to misfold. Installing robustness to anticodon mutation into our designs further reduced the number of tRNA sequences to 489 and 361 after successive iteration of the anticodon to UGA and CGA, respectively (Supplementary Fig. 13). Finally, the anticodon/variable ensemble of each tRNA was filtered such that any tRNA ensemble with an average diversity >8 Δbp or average frequency <50% was discarded, leaving 169 structurally robust tRNA sequences (Fig. 5e). These filtering thresholds were set so that 10^2^–10^3^ sequences were passed on to the next step.

Chi-T then clustered these 169 tRNA sequences, using affinity propagation, to give 33 cluster exemplars. From these 33 exemplars, a group of four tRNA sequences, which maximized the minimum Levenshtein distance between any pair in the four, was returned—that is, Chi-T picked the most diverse ensemble by ensuring that no two tRNAs were similar. The Levenshtein distance between any of the four tRNA sequences in the group was ≥18 bases (Fig. 5f).

Using RS-ID, we identified 80 potential tryptophanyl-tRNA synthetases (with cognate tRNAs bearing eight distinct sets of identity parts) to test with the tRNAs output by Chi-T (Supplementary Figs. 14 and 15). We tested three of these synthetases (*Eh*TrpRS, *Ph*TrpRS and *Tb*TrpRS), based on their known expression in *E. coli*^28,29^, with the four tRNAs that were output by Chi-T (Fig. 5g and Supplementary Fig. 16).

**Fig. 6:**
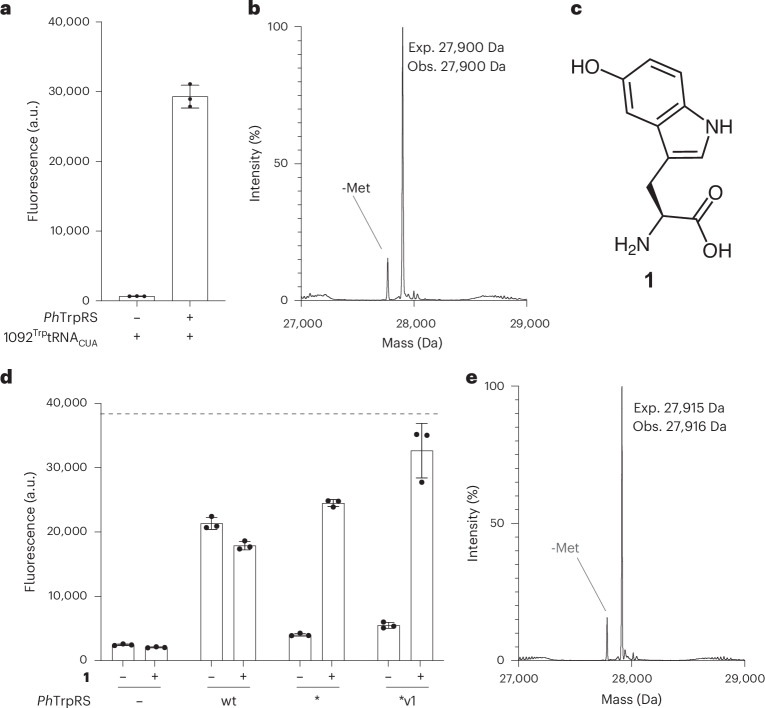
Characterizing a new orthogonal pair and engineering it for ncAA incorporation. **a**, GFP fluorescence from *sfGFP3TAG*_*His6*_ measured in cells containing 1092^Trp^tRNA_CUA_, with or without the *P. horikoshii* synthetase (*Ph*TrpRS). The experiments were performed in three independent replicates. The individual data points are shown as dots; the bars represent mean values; and the error bars indicate the standard deviation. **b**, Deconvoluted ESI-MS of GFP purified from cell containing *sfGFP150TAG*_*His6*_, 1092^Trp^tRNA_CUA_ and *Ph*TrpRS. Expected mass, sfGFP150Trp: 27,900 Da, observed 27,900 Da. The experiment was performed in one replicate. **c**, Structure of 5-hydroxy-l-tryptophan (**1**). **d**, GFP fluorescence from *sfGFP3TAG*_*His6*_ was measured in cells containing 1092^Trp^tRNA_CUA_ and the indicated *Ph*TrpRS variant in the presence and absence of **1** (Supplementary Fig. 18). The dashed line indicates the level of GFP fluorescence generated from *sfGFP3TAG*_*His6*_ by the *Mm*PylRS/*Mm*^Pyl^tRNA_CUA_ with 2 mM AllocK. The experiments were performed in three independent replicates. The individual data points are shown as dots; the bars represent mean values; and the error bars indicate the standard deviation. **e**, Deconvoluted ESI-MS of GFP purified from cell containing *sfGFP150TAG*_*His6*_, 1092^Trp^tRNA_CUA_, *Ph*TrpRS* and 5-hydroxy-l-tryptophan (**1**). Expected mass, GFP150Trp-OH: 27,915 Da, observed 27,916 Da. The experiment was performed in one replicate. Exp., expected; Obs., observed; wt, wild-type; v, variant. Source data

One of the tRNA sequences, 1092^Trp^tRNA_CUA_, produced low fluorescence from *sfGFP3TAG*, and addition of *Ph*TrpRS led to a 44-fold increase in GFP fluorescence; these experiments demonstrated that 1092^Trp^tRNA_CUA_ is an active and orthogonal tRNA (Fig. 6a). Mass spectrometry confirmed that the *Ph*TrpRS/1092^Trp^tRNA_CUA_ pair led to the selective incorporation of Trp in response to the amber codon in *sfGFP150TAG*_*His6*_ (Fig. 6b).

We also ran Chi-T with fixed identity parts derived from *Ph*^Trp^tRNA and *Tb*^Trp^tRNA and identified four tRNAs from each run. These tRNAs were not active with *Eh*TrpRS, *Ph*TrpRS and *Tb*TrpRS. Overall, we tested 12 chimeras in combination with three synthetases identified by RS-ID to discover an active and orthogonal tRNA.

We note that variants of 1092^Trp^tRNA_CUA_ in which the identity parts derived from *Eh*^Trp^tRNA were replaced with those from *Ph*^Trp^tRNA or *Tb*^Trp^tRNA were orthogonal and active with *Ph*TrpRS (Supplementary Fig. 16a,b). These experiments suggest that, as expected, the tRNA sequences generated by Chi-T are sensitive to the choice of starting identity parts but that orthogonal and active tRNAs identified with one set of identity parts may be functional with closely related sets of identity parts.

In additional experiments, we used Chi-T and RS-ID to generate an active and orthogonal 1081^Arg^tRNA_CUA_, which is aminoacylated by *Cs*ArgRS^17^. The starting point for discovering this pair was the identity parts from *Ft*^Arg^tRNA_CUA_; this tRNA did not fold into an MFE cloverleaf structure and was not active with *Ft*ArgRS or *Cs*ArgRS. To identify the active and orthogonal tRNA, we tested four chimeras with *Ft*^Arg^tRNA identity parts, in combination with two synthetases identified by RS-ID (Supplementary Fig. 17). These experiments demonstrated that Chi-T can be used to generate orthogonal and active tRNAs for an additional isoacceptor class.

The active and orthogonal tRNAs that we discovered via Chi-T contain 28 mutations (1092^Trp^tRNA_CUA_) and 14 mutations (1081^Arg^tRNA_CUA_) with respect to *Eh*^Trp^tRNA and *Ft*^Arg^tRNA. It would be exceptionally challenging to discover these sequences by directed evolution approaches, and we suggest that Chi-T enables the exploration of regions of sequence space not commonly accessible by other methods.

Overall, we discovered active and orthogonal tRNAs and cognate synthetases in one out of 36 combinations tested with identity parts from *Eh*^Trp^tRNA, *Ph*^Trp^tRNA and *Tb*^Trp^tRNA and in one out of eight combinations tested with *Ft*^Arg^tRNA identity parts, using Chi-T and RS-ID. We do not expect all combinations of identified synthetases and chimeric tRNAs to function, but we suggest that the sequence space sampled through Chi-T and RS-ID may be substantially enriched in orthogonal and active chimeric tRNAs and synthetases that may acylate them.

### ncAA incorporation with an orthogonal pair from Chi-T/RS-ID

To demonstrate that the *Ph*TrpRS/1092^Trp^tRNA_CUA_ pair can be engineered to incorporate ncAAs, we introduced mutations into the active site of *Ph*TrpRS (Y78F T79A I212G A214C), creating *Ph*TrpRS*. The analogous amino acid mutations in *Ec*TrpRS^30^ generate a variant that directs the incorporation of 5-hydroxy-l-tryptophan (**1**; Fig. 6c). Cells containing the *Ph*TrpRS*/1092^Trp^tRNA_CUA_ pair and *sfGFP150TAG*_*His6*_ exhibited low fluorescence in absence of **1** and an increase in fluorescence upon addition of **1** (Fig. 6d). Mass spectrometry confirmed that the *Ph*TrpRS*/1092^Trp^tRNA_CUA_ pair directs the incorporation of **1** into GFP (Fig. 6e).

To increase the activity of the *Ph*TrpRS*/1092^Trp^tRNA_CUA_ pair, we evolved the ACR of *Ph*TrpRS* (to accommodate the C35U mutation in 1092^Trp^tRNA_CUA_ with respect to *Ph*^Trp^tRNA_CCA_) (Supplementary Fig. 18). We identified a variant—*Ph*TrpRS*v1 (Y78F T79A I212G A214C T283H R286Q)/1092^Trp^tRNA_CUA_ pair—that exhibited a 30% increase in activity over the progenitor *Ph*TrpRS*/1092^Trp^tRNA_CUA_ pair. The *Ph*TrpRS*v1/1092^Trp^tRNA_CUA_ pair with 2 mM **1** produced approximately 80% of the fluorescence produced by the *Mm*PylRS/*Mm*^Pyl^tRNA_CUA_ with 2 mM AllocK, via read-through of the amber codon in *sfGFP150TAG*_*His6*_ (Fig. 6d). These experiments demonstrate that *Ph*TrpRS and its derivatives are orthogonal in *E. coli*.

In additional experiments, we used a *Ph*TrpRS*v1/1092^Trp^tRNA_CGA_ pair to decode TCG codons in Syn61Δ3ev5 *E. coli*. This is consistent with the Chi-T optimization of the 1092^Trp^tRNA cloverleaf structure with a CGA anticodon (Supplementary Fig. 19) and demonstrates that *Ph*TrpRS*v1 is tolerant to a single mutation in the anticodon of 1092^Trp^tRNA_CUA_. We also showed that the *Ph*TrpRS*v1/1092^Trp^tRNA_CGA_ pair is mutually orthogonal in its acylation specificity with respect to the widely used *Mm*PylRS/*Mm*^*Pyl*^tRNA_CUA_ pair and the four other pairs developed herein (Supplementary Fig. 20).

## Discussion

The MFE secondary structure prediction for many primary tRNA sequences shows little or weak cloverleaf folding, and, in their natural hosts, tRNAs may be stabilized by post-transcriptional modifications^19,20^ and other factors. Our analysis indicates that a common feature of orthogonal tRNAs is the ability of their unmodified primary sequence to fold into a robust predicted cloverleaf structure. We leveraged this insight to design active orthogonal tRNAs from inactive orthogonal tRNAs. Non-orthogonal tRNAs do not necessarily become orthogonal upon introducing mutations that favor a cloverleaf structure. However, we show that non-orthogonal tRNAs can be converted into active orthogonal tRNAs by targeted diversification of their sequences, using sequences from isoacceptors with minimal *E. coli* identity elements and filtering for predicted cloverleaf structures. These approaches allowed us to create orthogonal active tRNAs from starting points that were previously considered dead ends^17^.

We combined our insights into orthogonal tRNA generation to create Chi-T, an approach for the automated generation of candidate orthogonal tRNAs. Chi-T reduces the sequence space in which to search for orthogonal tRNAs from more than 4^76^ sequences to an experimentally testable number of sequences in four steps. The resulting chimeric tRNAs have distinct identity elements from *E. coli* tRNAs of the same isoacceptor class and minimal *E. coli* identity elements and are predicted to fold into cloverleaf structures. However, as parts of the tRNA sequence beyond identity elements clearly influence synthetase binding and acylation in ways that are poorly understood, there is no guarantee that any particular tRNA identified by Chi-T will be acylated by any particular synthetase that acylates other tRNAs with the same identity elements. To complement Chi-T, we developed RS-ID and used it to identify synthetases that recognize tRNAs with similar identity elements and identity parts to those in the chimeras generated by Chi-T. This allowed us to explore combinations of Chi-T-generated tRNAs, and synthetases that recognize the identity elements within those tRNAs, to discover active orthogonal tRNAs for two isoacceptor classes. We note that the synthetases that acylate orthogonal tRNAs are not necessarily orthogonal. We anticipate that Chi-T may be further scaled and may be refined by incorporating host-specific consensus sequences^31^, including sequences that direct EF-Tu binding^32^, or by defining the sites of post-transcriptional modification^33^. By specifying alternative host-specific identity elements^34^, it may also be possible to extend Chi-T to the scalable discovery of orthogonal tRNAs in diverse organisms.

## Methods

### Materials

Antibiotics and arabinose were obtained from Sigma-Aldrich. 5-Hydroxy-l-tryptophan was purchased from Acros Organics (cas. 4350-09-8, code 148290050). RNAfold webserver was used to create tRNA secondary structure illustrations^35^. A local installation of the ViennaRNA Package^18^ (version 2.4.18) was used to calculate all values given for the tRNA diversity and frequency. Molecular graphics and analyses were performed with UCSF Chimera, developed by the Resource for Biocomputing, Visualization, and Informatics at the University of California, San Francisco, with support from National Institutes of Health P41-GM103311 (ref. ^36^). GraphPad Prism version 9 was used to graph any collected data.

### Plasmid generation

These plasmids were obtained or adapted from the following: pKW1 (ref. ^17^) (spectinomycin resistance); p15A *CAT111TAG sfGFP150TAG*_*His6*_ (ref. ^17^) (tetracycline resistance); p15A *1R26*PylRS *CAT111TAG sfGFP150TAG*_*His6*_ (ref. ^15^) (tetracycline resistance); p15A *sfGFP3TAG*_*His6*_ (ref. ^3^) (apramycin resistance); and pKW1 *Methanosarcina mazei* pyrrolysine synthetase (*Mm*PylRS) with optimized ^Pyl^tRNA_CUA_ (refs. ^4,37,38^). Rationally designed tRNAs (*Ap*^His^tRNA_CUA_fix and *Cs*^Pro^tRNA_CUA_fix) were obtained by QuikChange mutagenesis using the respective pKW-(^Xxx^tRNA_CUA_) and pKW-(tRNA^X^)-aaRS as a template^39^. All Chi-T-generated tRNAs were obtained as duplex oligos (Integrated DNA Technologies (IDT)) with matching overhangs for ligation into a NotI/BglII (New England Biolabs (NEB), FastDigest) digested and gel-extracted pKW1 (tRNA^X^) vector. The coding sequences for each tryptophanyl synthetase (*Eh*TrpRS, *Tb*TrpRS and *Ph*TrpRS) were obtained as codon-optimized and recoded (Syn61) gBlocks (IDT) and inserted into a p15A vector containing *CAT111TAG* and *sfGFP150TAG*_*His6*_ by Gibson Assembly (NEB). All synthetase libraries were generated in the pKW-(^Xxx^tRNA_CUA_)-aaRS vector. The *Eh*TrpRS coding sequence was introduced into the pKW-1092^Trp^tRNA_CUA_ through Gibson Assembly^40^ (NEB). Mutants of *Eh*TrpRS were generated using a QuikChange protocol. ACR libraries were generated using Gibson Assembly or type IIS restriction digest (BsaI or SapI) and ligation (T4; NEB) of a polymerase chain reaction (PCR) product using degenerate primers targeting the residues of interest. For experiments in Syn61Δ3(ev5) (Addgene, bacterial strain 174514), derivatives of the p15A *sfGFP3 TCG4TAG*_*His6*_ (ref. ^3^) reporter plasmids (apramycin resistance; Addgene, 174518) and a recoded version of the pKW1 vector (spectinomycin resistance) were used. Plasmid and oligonucleotide sequences are listed in Supplementary Data 1.

### RNAfold analysis of tRNAs

All tRNA sequences for which tREX measurements were available were collected and folded using a local installation of RNAfold (version 2.4.18)^18,35^. *E. coli* K12 tRNA sequences were obtained from the Genomic tRNA Database (GtRNAdb)^41^. For all sequences, the MFE structure and its diversity and frequency were determined using the default settings of RNAfold. By manual inspection of the MFE structure, we assessed whether a sequence showed a cloverleaf fold or not. Small deviations from the theoretical optimal structure were accepted as long as the overall structure showed a clearly defined tRNA acceptor stem, D-arm, T-arm and anticodon loop, with the anticodon unpaired. Examples of small changes that were accepted include additional base pairing within a loop, pairing with the variable loop or base 8–9 and changes in line with the cloverleaf structure of tRNAs other than the predicted class. Not accepted were mismatches in the tRNA stem and any pairings between main tRNA domains (stem, D/L-arm or AC loop) or of the tRNA anticodon. All tRNAs judged to be compliant with these criteria were labeled as predicted cloverleaf tRNA (cloverleaf: yes), whereas any other structure is referred to as a non-cloverleaf tRNA (cloverleaf: no). The source data can be found in Supplementary Tables 4 and 5.

### Rational design of structurally fixed tRNAs

To stabilize the tRNA structure, we targeted elements of the tRNA body that do not carry identity elements—for example, the variable loop or anticodon stem. We aimed to use these mutations to generate unambiguous cloverleaf folds and minimize the possibility of other structures. The RNAfold prediction for the diversity and frequency was used as a measure of structural ambiguity, and we aimed to find high-frequency/low-diversity cloverleaf structures. In most cases, we found an unambiguous, folded cloverleaf tRNA by introducing 2–3-bp changes.

### Quantification of amber decoding

Next, 20 μl of chemically competent *E. coli* DH10B or Syn61Δ3(ev5) containing a transformed p15A GFP reporter plasmid (*sfGFP150TAG*_*His6*_ *CAT111TAG*, tetracycline resistant or *sfGFP3TAG*_*His6*_ apramycin resistant) was transformed with the pKW1 plasmid (conferring spectinomycin resistance) containing only the tRNA or the tRNA and its cognate synthetase. Syn61Δ3(ev5) was transformed with the reporter plasmid, followed by the pKW1 plasmid. Cells were recovered in 200 μl of SOB medium, 1,000 r.p.m., 1 h, 37 °C, before transfer into 2 ml of LB medium containing the appropriate antibiotics for selection. Cells were grown overnight (48 h for Syn61Δ3(ev5)) at 37 °C, 220 r.p.m. Then, 50 μl of each culture was diluted in triplicate into 450 μl of LB medium containing 0.2% L-arabinose and the appropriate antibiotics. Cultures were grown in a 96-well plate with 1.2-ml wells. For synthetases that recognize a non-canonical amino acid, a second triplicate expression containing 2 mM non-canonical amino acid (AllocK for *Mm*PylRS or 5-hydroxy-l-tryptophan for *Ph*TrpRS* and its variants) was performed. The wells were sealed with air-permeable foil, and the plate was incubated for 20 h in a shaking incubator at 37 °C, 1,000 r.p.m. Cells were then harvested by centrifugation (3,000*g*, 10 min); the medium was discarded; and the inverted plate was briefly placed on paper towels (1–2 min). The cell pellets were resuspended in 150 μl of PBS, of which 100 μl of resuspended cells was transferred to a clear, flat-bottom, 96-well plate (Nunc96). The optical density at 600 nm (OD_600_) and GFP fluorescence of each well were measured with a PHERAstar FS (BMG Labtech) plate reader. OD_600_ was measured using a 600-nm light source, and GFP fluorescence was measured using an optical module with an excitation wavelength of 485 nm and an emission wavelength of 520 nm (gain was set to 0). Plots show the average GFP fluorescence normalized by OD_600_ as a bar graph; the individual data points are shown as dots; and the standard deviation of the triplicate measurement is shown.

### Screening of Chi-T-generated tRNAs by GFP expression

Chemically competent DH10B cells were doubly transformed by heat shock (42 °C, 45 s, 20 μl of cells) with the pKW1-tRNA plasmid and p15A-synthetase GFP reporter plasmid. Cells were recovered for 1 h (200 μl of SOC medium, 37 °C, 850 r.p.m.). Each transformation was added to a different well of a 96-well plate (Nunc96, 1.2 ml or 2.2 ml) and diluted to 1 ml in LB containing tetracycline (10 μg ml^−1^) and spectinomycin (50 μg ml^−1^) and grown overnight (37 °C, 300 r.p.m.). Overnight cultures were diluted 10-fold into 450 μl of LB containing selection antibiotics and 0.2% L-arabinose to induce GFP expression and incubated at 37 °C (20 h). Measurements were performed as described above, and heatmaps show individual measurements of the transformed pool.

### GFP purification and mass spectrometry

Expression of sfGFP-His_6_ for purification was identical to those used during the tRNA screening. Expression was scaled to larger volumes (0.5 ml to 10 ml of LB) if necessary, and non-canonical amino acids were added at 2 mM. Cells were harvested by centrifugation, and the cell pellets were used directly for purification or stored frozen at −20 °C. The pellet was resuspended in 1 ml of 20 mM Tris-HCl pH 8, 150 mM NaCl containing 1× BugBuster Protein Extraction Reagent and lysed by agitating for 10 min at room temperature. The lysate was cleared by centrifugation (10 min, 15,000*g*), and Ni-NTA beads (20 µl of slurry) were added to the supernatant. The slurry was incubated while agitated for 1 h (room temperature). The beads were collected using a fritted spin filter (300*g*, 10 s) and washed three times with 1 ml of wash buffer (20 mM Tris-HCl pH 8, 150 mM NaCl, 40 mM imidazole). GFP was eluted in 50 µl of elution buffer (20 mM Tris-HCl pH 8, 150 mM NaCl, 200 mM imidazole). The elution buffer was exchanged for 20 mM Tris-HCl pH 8, 150 mM NaCl using a 10-kDa spin concentrator. High-resolution mass spectra of GFP were obtained by electrospray ionization mass spectrometry (ESI-MS) using a Waters Xevo G2 MS with a modified nanoAcquity LC system, as previously reported^3,17^. In brief, injected proteins were separated on a BEH C4 UPLC column (1.7 μm; 1.0 × 100 mm; Waters) with a flow rate of 50 μl min^−1^ using an acetonitrile gradient starting at 2% v/v to 80% v/v (0.1% v/v formic acid) over 20 min. The column outlet was directly interfaced via an ESI source with a hybrid quadrupole time-of-flight mass spectrometer (Waters). A cone voltage of 30 V was used during data acquisition in positive ion mode with a range of 300–2,000 *m*/*z*. The scans were deconvoluted using the MaxEnt1 function within MassLynx software (Waters). Spectra were also obtained using an Agilent 1200 liquid chromatography–mass spectrometry (LC–MS) system equipped with a 6130 Quadrupole spectrometer. Then, 10 μl of sample was applied on a Phenomenex Jupiter C4 column (150 × 2 mm, 5 μm), and a gradient of Buffer A (0.2% formic acid in water) and Buffer B (0.2% formic acid in acetonitrile (MeCN)) was used for reverse-phase high-performance liquid chromatography (HPLC), 10% to 90% B in 6 min. Mass spectra were acquired in positive mode and analyzed with MS ChemStation software (Agilent Technologies). The deconvolution program provided in the software was used to obtain the entire mass spectra. The expected, theoretical mass was calculated using ProtParam (Expasy) and adapted for the mass difference expected for a given non-canonical amino acid and GFP maturation.

### ACR selections

The ACR libraries were transformed into freshly made electrocompetent cells. The electrocompetent cells were prepared from an overnight culture of DH10B containing the p15A- *CAT111TAG* -*sfGFP150TAG*_*His6*_ selection plasmid. The overnight culture was diluted 1:100 in LB containing 10 μg ml^−1^ tetracycline and grown to OD 0.3 to 0.5 at 37 °C. The cells were harvested by centrifugation and washed three times with ice-cold water. After the final wash, all cells were resuspended in 200 μl of water and electroporated with 4 µg of the ACR library plasmid (4 × 1 µg in 50 μl of competent cells) using standard conditions for 2-mm electroporation cuvettes (2,500 V). One milliliter of SOB medium was added immediately after electroporation, and the cells were left shaking at 37 °C for 1 h to recover. A dilution series (10^−4^ to 10^−7^) was plated on selective plates to investigate library coverage (>10^8^ transformants). All cells were grown overnight and diluted 1:20 into selective media (20 ml, 2 mM **1** added for *Ph*TrpRS*) on the following day. Once cells reached exponential phase (OD_600_ > 0.5), they were harvested, and approximately 10^8^ cells were plated onto Cm selection plates (150 μg ml^−1^ chloramphenicol, 0.2% arabinose, 50 μg ml^−1^ spectinomycin, 10 µg ml^−1^ tetracycline and 2 mM **1** in case of *Ph*TrpRS*, 25 × 25 cm). The plates were incubated at 37 °C for 20 h. Generally, plasmid was isolated from colonies and retransformed into chemically competent DH10B containing the p15A *sfGFP3TAG*_*His6*_ (apramycin resistance), and GFP expression was tested. For all clones showing enhanced activity, the plasmid was isolated again from the retransformed colonies and sequenced to confirm that the orthogonal tRNA was intact and identify mutations in the synthetase. The order of these steps was changed depending on the selection outcome. Clones obtained from the *Ph*TrpRS* ACR selection were pre-screened for dependence of the GFP expression on **1** before plasmid isolation due to the large number of escape mutants via tRNA mutation (Supplementary Figs. 6, 7 and 18).

### Chi-T

Chi-T version 1 and version 1.1 were written using Python 3.7 to generate, filter and select chimeric tRNAs, which are publicly available in a GitHub repository (https://github.com/JWChin-Lab/). The following subsections were implemented within Chi-T: ‘tRNA database processing’, ‘Part scoring and selection and chimeric tRNA generation’, ‘Scoring and selecting chimeric tRNAs with minimal host identity elements’, ‘Folding and cloverleaf MFE structure-based filtering’ and ‘Chimeric tRNA clustering and selection’. Chi-T version 1.1 builds on version 1 by adding the ability to iterate processes as described in ‘Part scoring and selection and chimeric tRNA generation’.

#### tRNA database processing

Approximately 10,000,000 aligned tRNAs from bacteria, archaea, plants, fungi, viruses, phage, plasmids and chloroplasts were downloaded from tRNADB-CE^27^ (http://trna.ie.niigata-u.ac.jp/cgi-bin/trnadb/index.cgi). tRNA sequences in this database have been aligned and sectioned into the canonical structural parts. These tRNAs were cleaned using the cleanup.py script provided in Chi-T. This script removed any entries with missing information and then attempted to align D-loop sequences (because D-loops are variable in size, the common GG motif was used to align them). First, D-loop sequences from the tRNADB-CE dataset were checked against a manually curated D-loop alignment dictionary^17^. Those not in the dictionary were aligned automatically as follows. Shorter sequences (*n* = 6 or 7) were extended to the consensus 8 by adding two hyphens (‘--’) between the 2nd and 3rd (*n* = 6) nucleotides or one hyphen (‘-’) between the 3rd and 4th (*n* = 7) nucleotides. Then, sequences were searched for a ‘XXXGGX’ string (where X is any nucleotide or ‘-’), and sequences were aligned such that the GG motif comprised the 5th and 6th nucleotides in the D-loop (tRNA consensus nucleotides 18 and 19).

Nucleotides 74–76 were replaced with CCA, and the anticodon was replaced with CTA by default. Finally, sequences were merged into parts—for example, the D-loop (nucleotides 14–21, for example AACTGGCA) and the T-loop (nucleotides 54–60, for example TTCGAGC) were merged into a single part (AACTGGCA_TTCGAGC). Nine parts were used for chimera generation defined in Supplementary Fig. 9. Additional sequences (for example, from *E. histolytica*) were added using the tRNA_adder.py script provided in Chi-T.

#### Part scoring and selection and chimeric tRNA generation

tRNA sequences were filtered to remove those for isoacceptors other than the one specified, for example, to generate tRNAs using a tryptophanyl-tRNA synthetase as a target, the cleaned tRNA dataset was filtered by including only entries from natural tryptophanyl-tRNAs. At this point, identity parts and variable parts were defined based on the identity element positions for the given isoacceptor. All sequence parts were scored individually. For a single query sequence part, there are *k* positions that are identity elements for at least one isoacceptor. These positions are contained in the set *j*. For instance, a D-loop/T-loop part comprises positions 14–21 and 54–60 and may have the sequence AACTGGCA_TTCGAGC. Positions 14 (Leu), 15 (Cys, Leu, Pro), 16 (Leu), 20 (Ala, Arg, Phe), 59 (Phe) and 60 (Phe) are identity elements for at least one isoacceptor (specified in brackets)—therefore, *k* = *6* and the set *j* = {14, 15, 16, 20, 59, 60}. The identity of the nucleotide in the *E. coli* tRNA at position *i* (where *i* ∈ *j*) for isoacceptor *s* is *m*_*is*_, and so, at each position *i*, we define a multiset *M*_*i*_ containing the base identities of all the relevant isoacceptors from *E. coli* tRNAs. The corresponding nucleotide identity of the query part at position *i* is given as *n*_*i*_. Following on from the example above, the base identity of position 20 in the query sequence is C (*n*_*20*_ = *C*). The multiset *M*_*20*_ will contain *m*_*20A*_, *m*_*20R*_ and *m*_*20F*_ (the 20th nucleotide in the *E. coli* tRNAs for alanine, arginine and phenylalanine). In *E. coli*, these correspond to G, A and T, respectively, and so the multiset *M*_*20*_ is {G, A, T}. In *E. coli*, there are two alanine and four arginine tRNAs; however, the 20th nucleotide is G and A, respectively, in all tRNAs of the same isoacceptor. In cases where tRNAs of the same isoacceptor differ at an identity element, all unique base identities are counted once. The score at a single position *i* is defined as the number of times *n*_*i*_ appears in the multiset *M*_*i*_, divided by the size of *M*_*i*_. Because C appears zero times in *M*_*20*_ (${\sum }_{{m}_{20}\in {M}_{20}}{1}_{\{{m}_{20}=C\}}=0$), the score assigned to *n*_*20*_ is ${\sum }_{{m}_{20}\in {M}_{20}}{1}_{\{{m}_{20}=C\}}=0$/|*M*_*20*_| = 0/3 = 0. The overall part identity score for the query part sequence is the average score at each position in *j*, so that no element bears any more weight than any other element, because the relative importance of various identity elements to synthetase recognition is currently unknown. This scoring can be summarized in the following formula:

$$PartIdentityScore=\frac{1}{k}\sum _{i\in j}\frac{\left({\sum }_{{m}_{is}\in {M}_{i}}1\{{m}_{is}={n}_{i}\}\right)}{|{M}_{i}|}$$

For each part type, the lowest-scoring sequences were chosen for clustering (default number of sequences in Chi-T is 200). Affinity propagation clustering was performed using the Affinity Propagation function from the sklearn package on the sequence distance matrix, defined as the pairwise Levenshtein distances between sequences, for each variable part type (excluding the six sequences from the unpaired bases 8–9 part). Cluster exemplars were chosen by the clustering algorithm for each part type, and chimeric tRNAs were generated by combining all exemplar sequences in a given part type with all other exemplar sequences in every other part type (that is, the Cartesian product of all exemplars across part types).

In Chi-T version 1.1, there is an option to iterate the process of chimeric tRNA generation and structural filtering to generate more diverse tRNAs. In this approach, the variable parts selected for chimeric tRNA generation (cluster exemplars) are excluded from the parts used for subsequent iterations in which revised sets of parts are clustered and new cluster exemplars are chosen to proceed with. This process may be repeated for several iterations.

#### Scoring and selecting chimeric tRNAs with minimal host identity elements

Assembled chimeric tRNAs were first filtered by length (tRNAs <79 nt were kept) and then scored by their sequence identity^17^. Rather than one aggregate score across all isoacceptors, as in the part identity score, a sequence identity score consists of one score for each isoacceptor, and the highest score for any isoacceptor is taken for filtering. In brief, to score a query tRNA sequence for a single isoacceptor—for example, alanine—for each nucleotide in the sequence that is an identity element of alanine (nucleotides 2, 3, 4, 20, 69, 70, 71 and 73), the alanine score is increased by 1 if the query sequence nucleotide matches the *E. coli* sequence and is decreased by 1 if it does not. This cumulative total is then divided by the number of elements (eight in this case). For isoacceptors with multiple tRNAs, the scoring is performed for each *E. coli* tRNA and then averaged to give an isoacceptor score. This process is repeated for all isoacceptors. Here, the scoring was modified such that identity elements within identity parts specific for a given Chi-T run were not involved in scoring, as these sequences were fixed throughout the process. All remaining chimeric sequences were scored, and the lowest-scoring tRNAs were put forward for folding and cloverleaf MFE structure-based filtering.

#### Folding and cloverleaf MFE structure-based filtering

All tRNAs were computationally folded using RNAfold, using the default parameters for MFE structure production. MFE structures were filtered based on their structure, frequency and ensemble diversity. The secondary structure string representation was used for filtering according to the regular expression string provided in Supplementary Fig. 9. Sequences that formed cloverleaf tRNAs but whose structures did not correspond to their parts—for example, where a D-loop has partially paired into the D-arm—were accepted, provided there was at least one thymine/uracil in the first unpaired section (unpaired bases 8–9) and the most 5′ nucleotide of the D-loop was an adenine. tRNAs passing structural filtering for their individual tRNA sequences were further filtered based on the average structural metrics of their anticodon/variable ensemble (Supplementary Fig. 13) with stricter thresholds.

#### Chimeric tRNA clustering and selection

The output from folding and cloverleaf MFE structure-based filtering was optionally filtered by sequence identity to the user-defined tRNA before implementing the clustering described in this subsection. In this option, tRNAs with more mismatches to the given reference tRNA than the user-defined threshold were discarded. When run in automatic mode, Chi-T aims to select 200 sequences for clustering, achieved by iteratively removing sequences with the largest number of mismatches from the parent tRNA, until fewer than 200 sequences remain.

tRNA sequences passing all previous filters were clustered using the same function and parameters for part sequences above, and the cluster exemplar sequences were taken forward. The group of output tRNAs was chosen from this pool as having the highest minimum Levenshtein distance between any pair in the group (Chi-T default is four tRNAs).

### Parameters used for generating chimeric tRNAs through Chi-T

^Trp^trRNA_CUA_ chimeras were generated in Chi-T version 1 with the following settings and parameters. We specified what the target ^Trp^tRNA sequence is by its tRNADB-CE ID (tRNAs not in the database can be added using the tRNA_adder.py script for Chi-T), and target isoacceptor class was set to Trp to extract all tryptophanyl-tRNA for part scoring. For ‘Part scoring and selection and chimeric tRNA generation’, the top scoring 65 part sequences for each part type were clustered to generate chimeric tRNAs from (--cluster_parts65). For ‘Scoring and selecting chimeric tRNAs with minimal host identity elements’, tRNAs 79 nt or longer were discarded (length_filt 79) and the following parameters for minimizing host identity were used: (cervettini_filt 0.5 (starting_stringency), 0 (minimum_stringency), 2,500,000 (target number of tRNAs), 0.05 (step size)); this starts with an identity score of no more than 0.5 (for any host synthetase) and reduces the identity score used to filter until 2.5 million sequences are left.

‘Folding and cloverleaf MFE structure-based filtering’ was done over three anticodons—CTA, TGA and CGA—with a frequency cutoff of 40% and a diversity cutoff of 9 Δbp for each individual tRNA sequence (--anticodons CTATGA CGA; --frequency 0.4; --diversity 9). To filter the anticodon varied ensemble an average frequency threshold of 50% (final_frequency 0.5) and an average_diversity threshold of 8 Δbp (--final_diversity 8) were set. In ‘Chimeric tRNA clustering and selection’, the automatic mode was used (--automatic); it was not necessary to filter out tRNA sequences to have fewer than 200 sequences for clustering. In the remaining tRNAs, the set of four most distant tRNAs from the cluster exemplars were searched (--num_tRNAs 4).

^Arg^tRNA_CUA_ chimeras were generated in Chi-T version 1.1 with the following settings and parameters. The target *Ft*^Arg^tRNA sequence is specified by its tRNADB-CE ID and target issoacceptor class to Arg to extract all arginyl-tRNA for part scoring. For ‘Part scoring and selection and chimeric tRNA generation’, the top 60 part sequences for each part type were clustered to generate chimeric tRNAs from (--cluster_parts 60). Chimeric tRNA generation was run in three iterations each excluding the exemplar parts used in all previous iterations (--num_iterations 3). For ‘Scoring and selecting chimeric tRNAs with minimal host identity elements’, tRNAs 79 nt or longer were discarded (--length_filt 79), and the following parameters for minimizing host identity were used: (--cervettini_filt 0.5 (starting_stringency), 0 (minimum_stringency), 2,500,000 (target number of tRNAs) and 0.05 (step size)). ‘Folding and cloverleaf MFE structure-based filtering’ was done over seven anticodons—CTA, TGA, CGA, TGC, CGC, AGA and GGA—with a frequency cutoff of 30% and a diversity cutoff of 10 Δbp for each individual tRNA sequence (--anticodons CTA TGA CGA TGC CGC AGA GGA; --frequency 0.3; --diversity 10). To filter the anticodon varied ensemble, an average frequency threshold of 50% (--final_frequency 0.5) and an average diversity threshold of 3 Δbp (final_diversity 3) were set. In ‘Chimeric tRNA clustering and selection’, the automatic mode was used (--automatic). To get fewer than 200 sequences for clustering, sequences with more than 14 mismatches with respect to *Ft*^Arg^tRNA were filtered out. In the remaining tRNAs, the set of four most distant tRNAs from the cluster exemplars was searched (--num_tRNAs 4).

### Synthetase Identification with RS-ID

The inputs for RS-ID include (1) the processed database output of cleanup.py (above); (2) an isoacceptor class; (3) the name(s) of cognate synthetase to a tRNA(s) with the identity parts of interest; and, optionally, (4) the threshold for identity element mismatches and (5) sequence similarity threshold for identity parts.

Within RS-ID, the tRNA database is filtered for sequences in the specified isoacceptor class. The identity parts for the tRNAs are identified as well as the identity elements within them. All identity parts in a given tRNA are concatenated, and unique concatenated sequences are identified; these are termed ‘concatenated identity parts’. These concatenated identity parts are compared to the concatenated identity parts of the user-defined tRNA (or tRNAs). Concatenated identity parts with more than one mismatch (a user-defined parameter) in the identity element nucleotides, with respect to the user-defined tRNA(s), are discarded. The remaining concatenated identity parts are stored in FASTA format and input into the calc_distmx function, part of the USEARCH software suite (version 11)^42^, to generate a sequence dissimilarity matrix for all unique concatenated identity parts. Similar sequences to the user-defined concatenated identity parts are identified using a similarity threshold (default is 0.2, where 0 is identical and 1 is no homology) and returned. The matrix is converted into a two-dimensional projection using the uniform manifold approximation and projection (UMAP) algorithm^43^, and any clusters were identified using HDBSCAN^44^ on the UMAP embeddings. The outputs of RS-ID are a UMAP projection and a spreadsheet containing the remaining filtered tRNA gene IDs, their identity part sequences, any HDBSCAN-assigned cluster and the organisms from which remaining filtered tRNAs originate. The synthetase genes were then extracted from the genomes of the organisms output by RS-ID.

### Reporting summary

Further information on research design is available in the Nature Portfolio Reporting Summary linked to this article.

## Online content

Any methods, additional references, Nature Portfolio reporting summaries, source data, extended data, supplementary information, acknowledgements, peer review information; details of author contributions and competing interests; and statements of data and code availability are available at 10.1038/s41589-024-01782-3.

## Supplementary information

Supplementary Figs. 1–20 and Supplementary Note 1.

Reporting Summary

Supplementary Tables 1–6.

Supplementary Data 1 Plasmid and oligo sequences.

## Source data

Source Data Figs. 1–6 Contains the source data for all figures in a single file.

## Data Availability

Data supporting this study can be found within the article, Source Data, Supplementary Tables, Supplementary Data and Supplementary Information. Data for tRNAs were downloaded from https://trna.ie.niigata-u.ac.jp/cgi-bin/trnadb/index.cgi. Protein Data Bank entries used in this study include 1H4Q, 4RDX and 2AKE. Source data are provided with this paper.

## Code availability

The Chi-T (version 1 and version 1.1) code and RS-ID code, for computational design of chimeric orthogonal tRNA and for identification of organisms from which to test synthetases for the chimeric orthogonal tRNAs, are available at https://github.com/JWChin-Lab.
