# Supplementary material for: Automated orthogonal tRNA generation
Source: Nat Chem Biol. 2024 Dec 20;21(5):657–67. doi: 10.1038/s41589-024-01782-3 (PMC12037408; doi:10.1038/s41589-024-01782-3)
Supplement: Supplementary file 1 — Supplementary Figs. 1–20 and Supplementary Note 1. [file 41589_2024_1782_MOESM1_ESM.pdf]

nature chemical biology

Supplementary information https://doi.org/10.1038/s41589-024-01782-3

Automated orthogonal tRNA generation

In the format provided by the authors and unedited

Table of Contents

Supplementary Figure 1. Frequency-diversity plots of *E. coli* tRNAs and tRNAs previously characterized as U, NO, OI, OA. 2

Supplementary Figure 2. Rational design of Cs^{Pro}tRNA$_{CUA}$fix and Af^{His}tRNA$_{CUA}$fix. 3

Supplementary Figure 3. *Cs*ProRS/Cs^{Pro}tRNA$_{CUA}$fix directs the incorporation of proline in response to the amber codon. 5

Supplementary Figure 4. T2H6 ^{His}tRNA$_{CUA}$fix, T2H7 ^{His}tRNA$_{CUA}$fix and T2H9^{His}tRNA$_{CUA}$fix, when paired with *Ap*HisRS, direct the incorporation of histidine in response to the amber codon. 6

Supplementary Figure 5. MFE structure of T2H1 to T2H12, and the point mutations introduced to generate T2H1, T2H2, T2H10, T2H11, T2H12 from their precursors. 7

Supplementary Figure 6. Directed evolution of *Ap*HisRS to function with T2H9^{His}tRNA$_{CUA}$fix. 8

Supplementary Figure 7. Directed evolution of *Cs*ProRS to function with Cs^{Pro}tRNA$_{CUA}$fix. 10

Supplementary Figure 8. Part Identity Scoring methodology. 12

Supplementary Figure 9. Cloverleaf structure definition. 14

Supplementary Figure 10. RNAfold MFE structure of Mm^{Pyl}tRNA variants with different anticodons. 15

Supplementary Figure 11. Pseudocode of the RS-ID script. 16

Supplementary Figure 12. The reduction in part number through part selection. 17

Supplementary Figure 13. Filtering chimeric TrptRNACUA sequences with EhTrptRNA identity parts, to identify sequences that are robust to anticodon mutation. 18

Supplementary Figure 14. RS-ID script for Eh^{Trp}tRNA identity parts. 19

Supplementary Figure 15. Alignment of Chi-T input tRNAs and generated designs. 20

Supplementary Figure 16. Screening of Chi-T designed tRNAs, with identity parts from Tb^{Trp}tRNA, Eh^{Trp}tRNA and Ph^{Trp}tRNA, with synthetases identified by RS-ID. 21

Supplementary Figure 17. Generation of 1081^{Arg}tRNA$_{CUA}$, an active orthogonal arginyl-tRNA, by Chi-T. And, the RS-ID led identification of a synthetase (*Cs*ArgRS) that functions with 1081^{Arg}tRNA$_{CUA}$ 22

Supplementary Figure 18. Directed evolution of *Ph*TrpRS* anticodon recognition for 1092^{Trp}tRNA$_{CUA}$ 24

Supplementary Figure 19. Decoding activity of 1092^{Trp}tRNA anticodon variants in Syn61D3(ev5). 26

Supplementary Figure 20. Determining mutual orthogonality of the tRNA/-synthetases pairs reported in this work. 27

Supplementary Note 1. On native tRNA folding and activity. 28

References 29

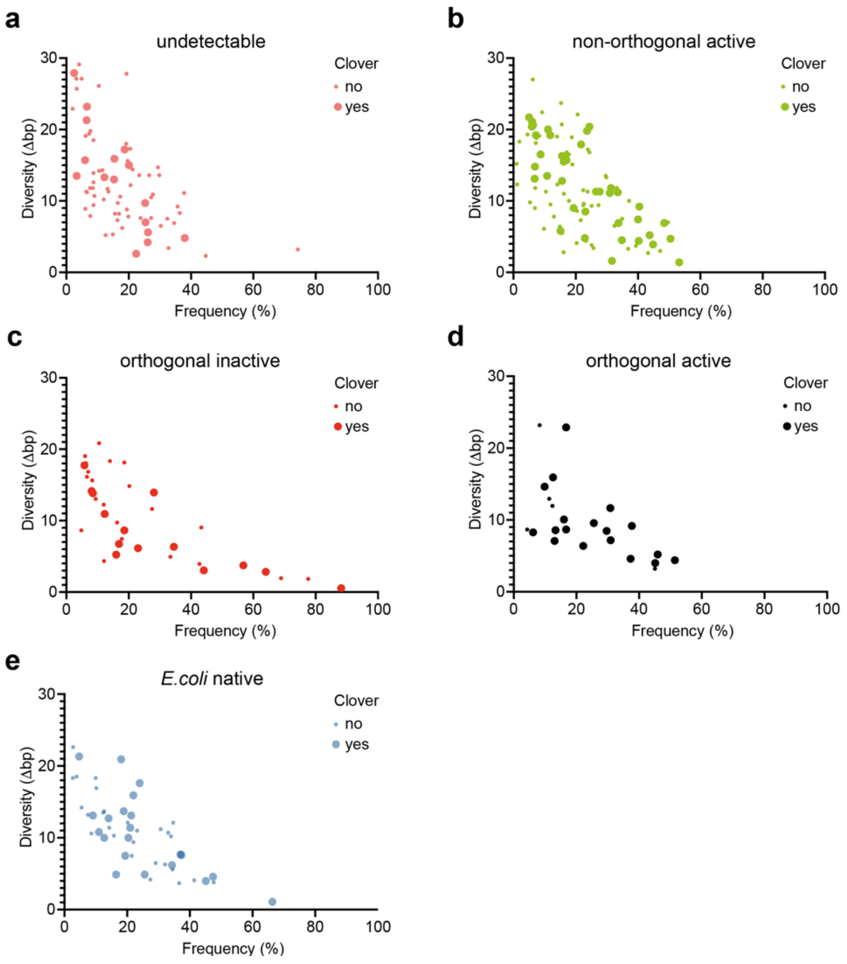

Supplementary Figure 1. Frequency-diversity plots of *E. coli* tRNAs and tRNAs previously characterized as U, NO, OI, OA. a-d) Predicted minimum free energy (MFE) structures for all 243 tRNAs in Cervettini et al. 2020 were calculated using RNAfold and plotted in the indicated groups. **e**) Predicted minimum free energy (MFE) structure of *E. coli* tRNAs from RNAfold. The plots show the frequency (%) and diversity (Δbp). Large circle indicates cloverleaf folding, other folds are depicted as small circles[1].

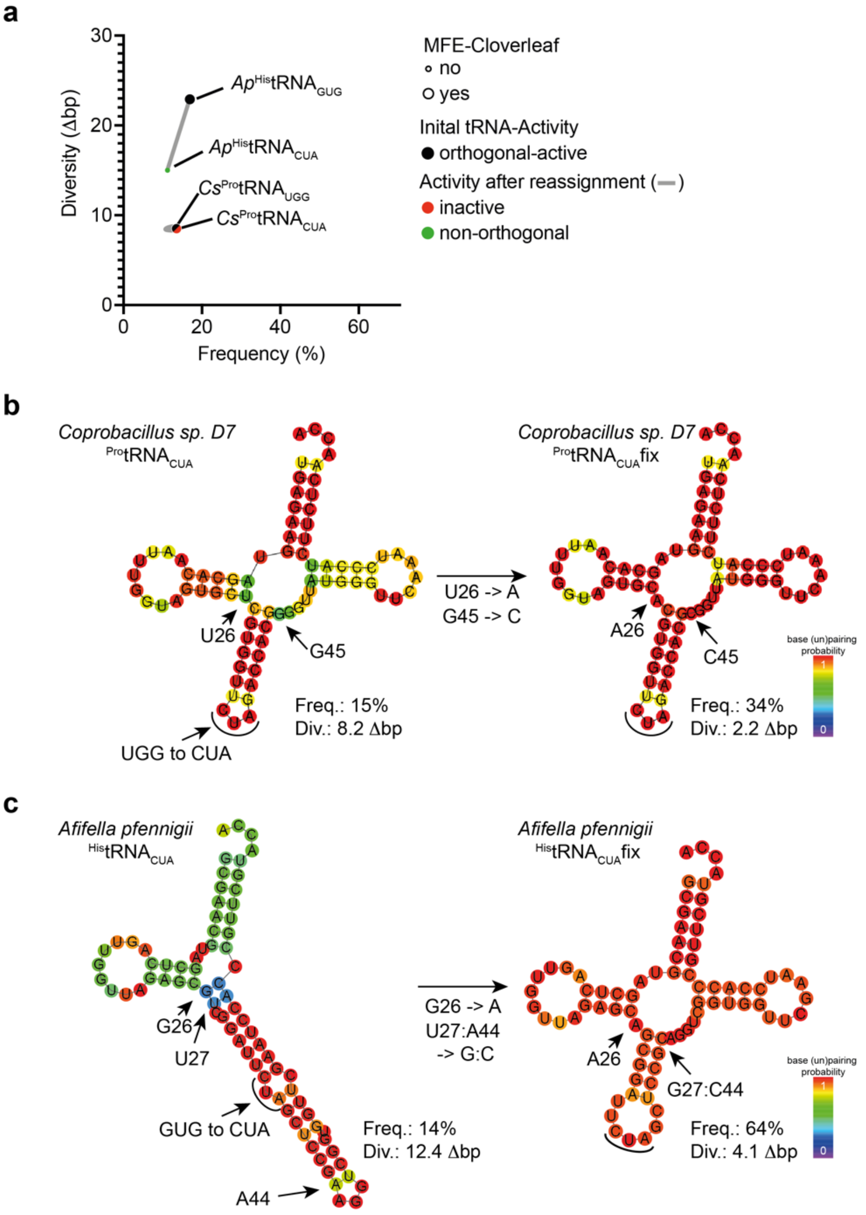

Supplementary Figure 2. Rational design of *Cs*^{Pro}tRNA$_{CUA}$fix and Af^{His}tRNA$_{CUA}$fix.

a) Effect of changing tRNA anticodons of *Ap*^{His}tRNA$_{GUG}$ and *Cs*^{Pro}tRNA$_{UGG}$ and CUA on RNA to frequency, diversity calculated in RNAfold. Native orthogonal-active tRNAs are depicted as black dots and the amber reassigned variant as coloured dots, the colour indicates whenever the tRNA became non-orthogonal (green) or inactive (red) following reassignment to amber. The native and amber reassigned tRNA are connected by a grey line. **b**) RNAfold predicted MFE structure, frequency (%) and diversity (Δbp) for the *Coprobacillus sp. D7* ^{Pro}tRNA$_{CUA}$. **c)** RNAfold predicted MFE structure, frequency (%) and diversity (Δbp) for *Afifella pfennigii*

$^{His}tRNA_{CUA}$. Arrows indicate the positions where mutations were introduced. tRNA anticodons indicated by a black arc. Colour gradient indicates the base (un) pairing probability for the depicted MFE structure.

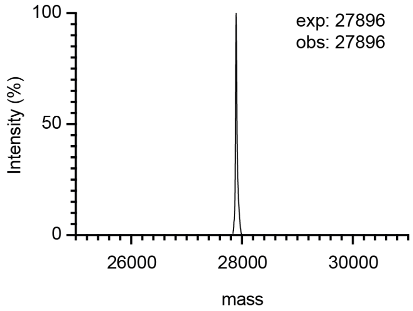

Supplementary Figure 3. *Cs*ProRS/*Cs*^{Pro}tRNA$_{CUA}$fix directs the incorporation of proline in response to the amber codon.

Electrospray ionization mass spectrometry of sfGFP purified from cells expressing *Cs*^{Pro}tRNA$_{CUA}$fix with *Cs*ProRS and *sfGFP3TAG*$_{His6}$. Expected mass of sfGFP-His6 incorporating proline at position 3: 27896 Da. Found mass 27896 Da.

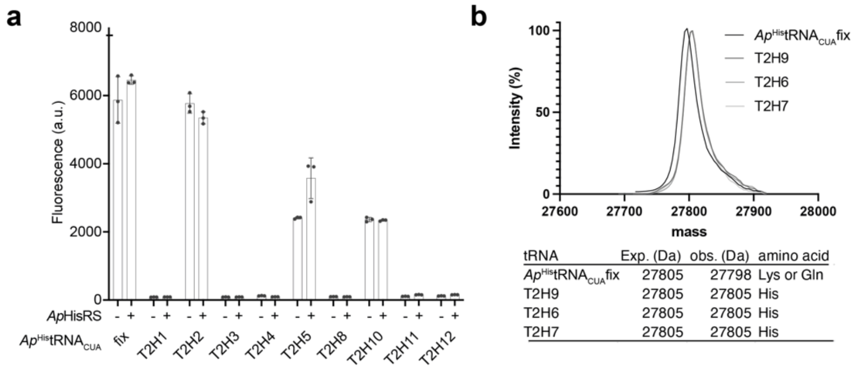

Supplementary Figure 4. T2H6 ^{His}tRNA$_{CUA}$fix, T2H7 ^{His}tRNA$_{CUA}$fix and T2H9^{His}tRNA$_{CUA}$fix, when paired with *Ap*HisRS, direct the incorporation of histidine in response to the amber codon.

a) GFP fluorescence from cells bearing *sfGFP3TAG$_{His6}$*, *Ap*HisRS and the indicated inactive or non-orthogonal *Ap*^{His}tRNAs (T2H1, T2H2, T2H3, T2H4, T2H5, T2H8, T2H10, T2H11, T2H12, *Ap*^{His}tRNA$_{CUA}$fix). The dots represent the measurement from three independent replicates, the bar shows the mean, and the error bars show the standard deviation from the mean. **b**) Electrospray ionization mass spectrometry of sfGFP-His6 purified from cells expressing *sfGFP3TAG$_{His6}$*, *Ap*^{His}tRNA$_{CUA}$fix or its derivatives (T2H6, T2H7 or T2H9) with *Ap*HisRS. Expected mass of sfGFP incorporating histidine in response to the amber codon in sfGFP3TAG$_{His6}$ is 27805 Da. Based on the mass difference to histidine, *Ap*^{His}tRNA$_{CUA}$fix directs the incorporation of lysine or glutamine, as its parental *Ap*^{His}tRNA$_{CUA}$ does[1]. T2H7, T2H8 and T2H9 direct the incorporation of histidine.

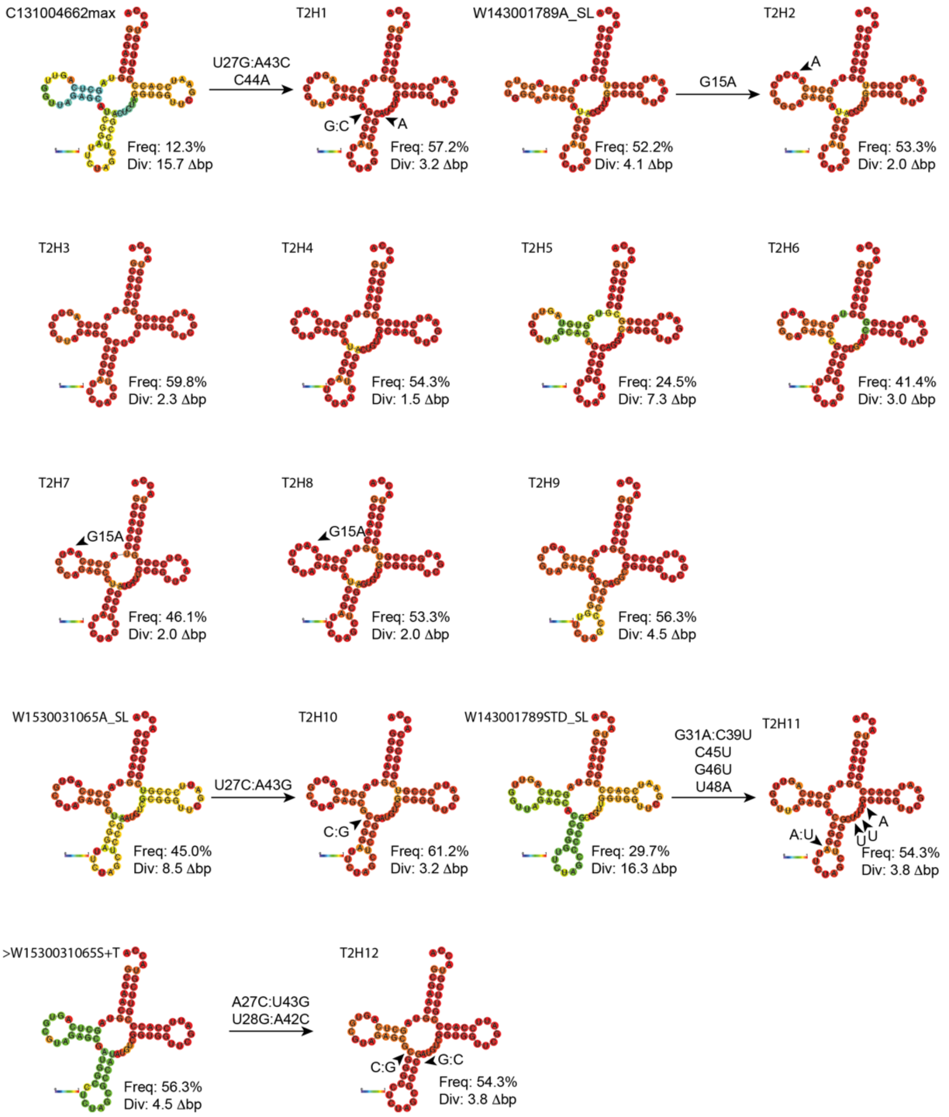

Supplementary Figure 5. MFE structure of T2H1 to T2H12, and the point mutations introduced to generate T2H1, T2H2, T2H10,T2H11,T2H12 from their precursors.

Sequence of the 12 selected chimeras between *Ap*^{His}tRNA$_{CUA}$fix and twelve other ^{His}tRNAs. Changes made to improve folding or remove potential identity elements with respect to the pre-cursor sequences are indicated. G15 is an identity element of some *E. coli* tRNA-synthetases, but the presence of G15 in T2H9 did not adversely impact its orthogonality. Arrows indicate the designed mutations. Base colour indicates the probability of the pairing status of each base as it appears in the MFE structure. The calculated MFE structures' frequency (%) and diversity (Δbp) is given.

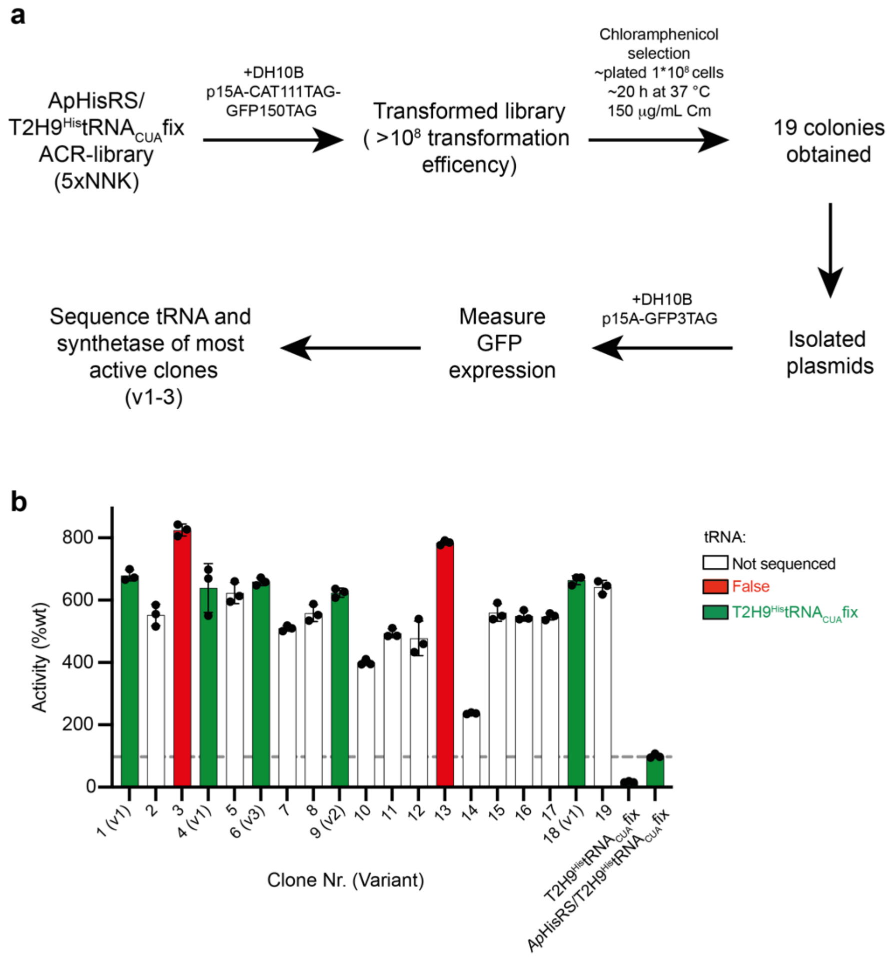

Supplementary Figure 6. Directed evolution of *Ap*HisRS to function with T2H9^{His}tRNA$_{CUA}$fix.

a) Selection procedure for the *Ap*HisRS anticodon recognition library. The anticodon recognition library was constructed by randomizing the codons for five residues (Ser482, Asp 483, Glu 484, Gly491 and Arg493) to NNK in the anticodon recognition domain of the *Ap*HisRS gene. Clones were obtained following selection for readthrough of an amber stop codon in the chloramphenicol acetyltransferase gene (*CAT111TAG*), and subsequently validated by *sfGFP3TAG*$_{His6}$ expression and sequencing of the tRNA and synthetase gene. **b)** Relative GFP fluorescence (from *sfGFP3TAG*$_{His6}$), normalised by OD$_{600}$, of cells containing only T2H9^{His}tRNA$_{CUA}$fix, the wild-type *Ap*HisRS/T2H9^{His}tRNA$_{CUA}$fix pair, or the indicated clone plasmid. Clone plasmids were isolated following chloramphenicol selection and

transformed into GFP reporter strain; the plasmids were expected to contain the genes for an *Ap*HisRS variant and the T2H9^{His}tRNA$_{CUA}$fix. For five clones the unchanged orthogonal T2H9^{His}tRNA$_{CUA}$fix gene was confirmed by sequencing (green), two clones did not contain T2H9^{His}tRNA$_{CUA}$fix (red) and were discarded, the remaining clones were not sequenced (white). The synthetase variant identified for clones, identified by sequencing, is shown in brackets. Overall, three unique *Ap*HisRS variants were identified. All measurements were performed in independent triplicates and normalized to the activity of the *Ap*HisRS/T2H9^{His}tRNA$_{CUA}$fix pair. The dots show the individual data points. The bars show the mean values and the error bars show the standard deviation from the mean.

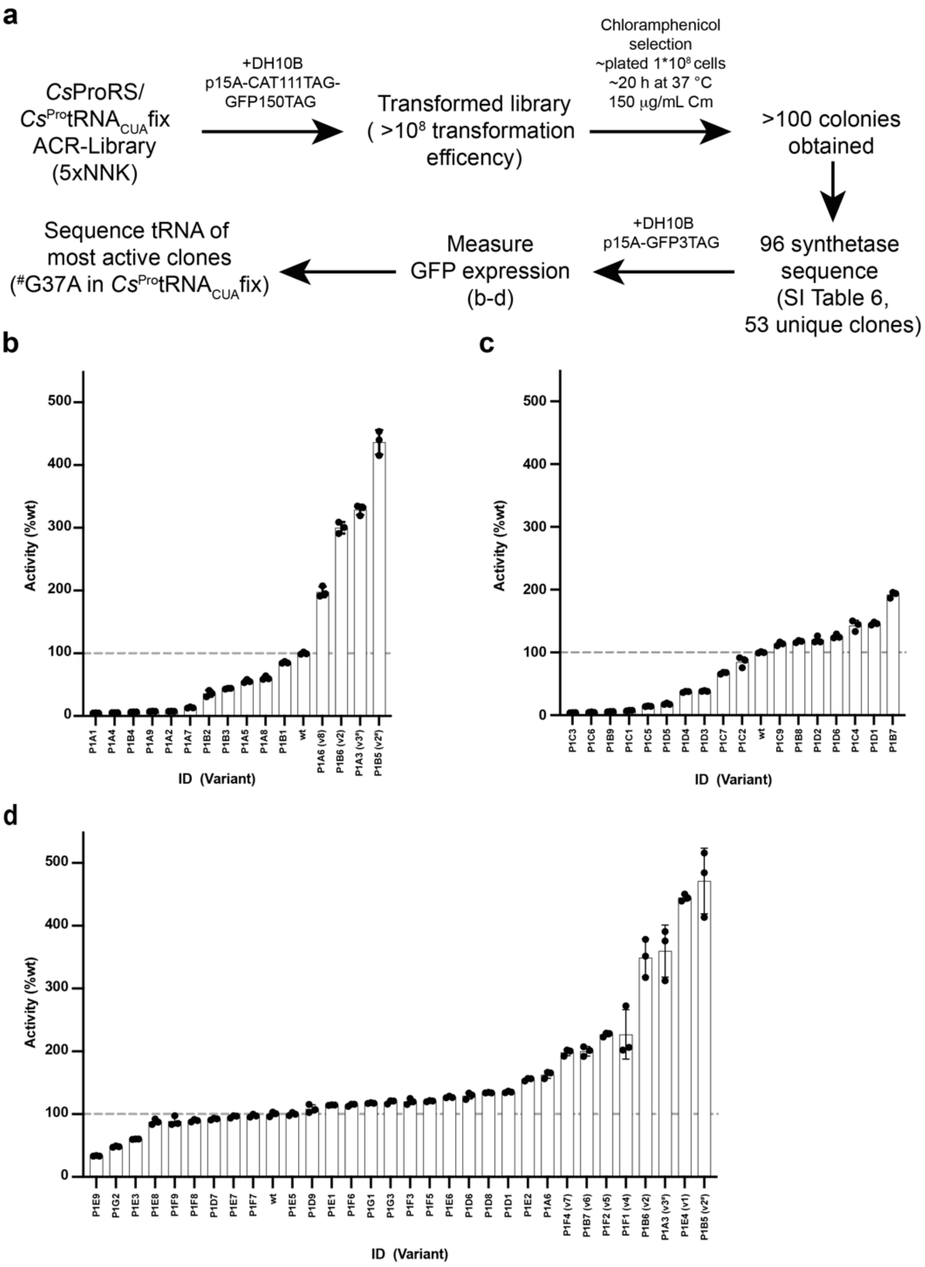

Supplementary Figure 7. Directed evolution of *Cs*ProRS to function with Cs^{Pro}tRNA$_{CUA}$fix.

a) Selection procedure. The library was constructed by randomizing the codons for five residues (Glu340, Arg347, Lys370, Glu349 and Asp354) to NNK in the anticodon recognition domain of the *Cs*ProRS gene. Clones were obtained following selection by readthrough of an amber stop codon in the chloramphenicol acetyltransferase gene (*CAT111TAG*) and subsequently validated by *sfGFP3TAG*$_{His6}$ expression (**b-d**) and sequencing of the tRNA and

synthetase genes. **b-d**) GFP fluorescence (*sfGFP3TAG*$_{His6}$) normalised by OD_{600} of cells containing the retransformed plasmid isolated from all 53 clones (**Supplementary Table 6**). The identified synthetase variant is shown in brackets. All measurements were performed in independent triplicates and normalized to the activity of the *Cs*ProRS/*Cs*^{Pro}tRNA$_{CUA}$fix pair. The dots show the individual data points. The bars show the mean values and the error bars show the standard deviation from the mean.

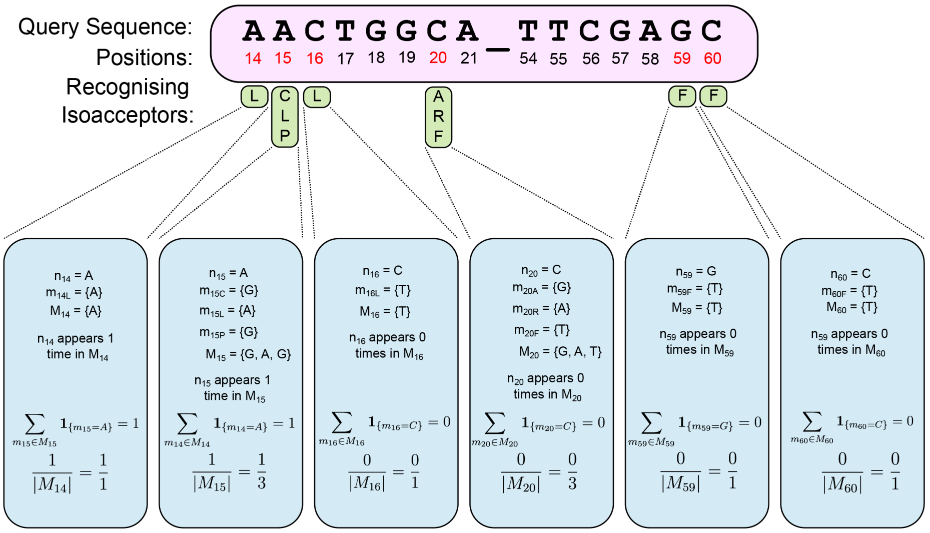

k=6

j={14, 15, 16, 20, 59, 60}

$$\frac{1}{k}\sum_{i\in j}\frac{\sum_{m_i\in M_i}\mathbf{1}_{\{m_i=n_i\}}}{|M_i|} = \frac{1}{6}\times(\frac{1}{1}+\frac{1}{3}+\frac{0}{1}+\frac{0}{3}+\frac{0}{1}+\frac{0}{1}) = 0.222$$

Supplementary Figure 8. Part Identity Scoring methodology.

A example of the Part Identity Scoring method used to identify variable parts for chimeric tRNAs. The query sequence AACTGGCA_TTCGAGC from the D-loop/T-loop part {14-21+54-60} contains positions 14, 15, 16, 17, 18, 19, 20, 54, 55, 56, 57, 58, 59, 60. The positions that have known identity elements for any isoacceptor are shown in red (14, 15, 16, 20, 59, 60), with the synthetase isoacceptor in question shown beneath e.g. position 15 is used for tRNA recognition as an identity element by cysteine (C), leucine (L), and proline (P) synthetases. The positions in red constitute the set *j*, whose size is given by *k*. In this case, *j* = {14, 15, 16, 20, 59, 60) and so *k* = 6. For each position, the relevant calculations are shown. Using position 15 as an example, the base identity of the query sequence at position 15 (n_{15}) is A. The base identities for the *E. coli* cysteine tRNA at position 15 is G, and for proline is also G. The six leucine tRNAs in *E. coli* all have A at position 15, and so A is counted once (m_{15A} = {A}, rather than {A, A, A, A, A, A}.) The multiset M_{15} is the concatenation of all these sets, {G, A, G}. The indicator function $\mathbf{1}_{\{m15=A\}}$ equals 1 if m_{15} = A, and 0 if not. Summing the indicator function

across all the elements of M_{15} is therefore 1 (A appears once in {G, A, G}. This is divided by the size of M_{15}, and so the contribution to the part score at position 15 is 1/3. This scoring is repeated at every position in *j*, and the final score is the average at each position, shown in the example by dividing the sum of each score by the number of positions, *k*.

a

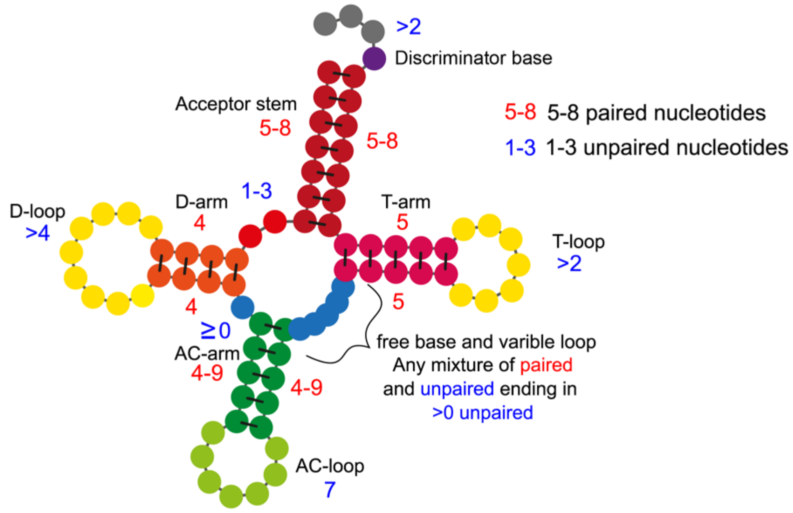

b

```
MFE string (example tRNA):
(((((((( .. (((( ......... )))) . ((((( ....... ))))) ..... ((((( ...... ))))) )))))))) ....
Base number variation:
  5-8   1-3   4    >4        4  ≥0  4-9     7     4-9    >0    5     >2     5     5-8    >2
Allowed (un)paired
   (     .    (     .        )   .   (       .      )  .+.()  (      .     )      )      .
Univerasal clover-leaf tRNA search pattern:
^\\({5,8}\\.{1,3}\\({4}\\.{5,}\\){4}\\.*\\({4,9}\\.{7}\\){4,9}.*\\.\\({5}\\.{3,}\\){5}\\)
{5,8}\\.{3,}$
```

Supplementary Figure 9. Cloverleaf structure definition.

a) The canonical tRNA cloverleaf secondary structure, with part types distinguished by colour. Here, blue describes the required number of unpaired bases, and red describes the number of paired bases (‘Base number variation’ and ‘allowed (un)paired)’. The variable loop can take a number of structures featuring paired and unpaired bases, only requiring a minimum of one unpaired base at the 3’ end of the part i.e. an unpaired base must separate the anticodon stem and the T-arm. **b)** RNAfold outputs’ MFE structures in dot-bracket secondary structure format, such that unpaired bases are denoted by ‘.’, and paired bases are denoted by ‘(‘ or ‘)’, depending on if they are pairing with bases 3’, or 5’ of themselves, respectively (‘MFE string’). We defined a cloverleaf structure based on the order and number of dots and brackets. We searched for these structures using the regex string (‘Universal cloverleaf tRNA search pattern’). All cloverleaf structures are also defined by the first continuous unpaired region containing at least one uracil (unpaired bases 8-9 in canonical structure), and the first nucleotide of the second continuous unpaired region being an adenine (nucleotide 14, D-loop, in canonical structure).

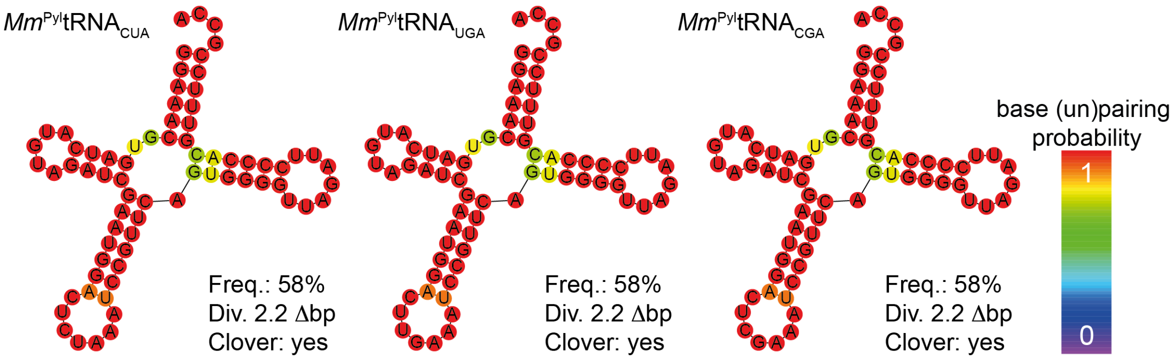

Supplementary Figure 10. RNAfold MFE structure of *Mm*^{Pyl}tRNA variants with different anticodons.

RNAfold predicted minimum free energy structure of *Mm*^{Pyl}tRNA$_{\mathrm{CUA}}$, and its UGA and CGA anticodon variants. The base colour indicates the probability of each base to pair as predicted in their MFE structure and the calculated MFE structures' frequency (%), diversity (Δbp) and cloverleaf classification (yes/no) is given. The activity of *Mm*^{Pyl}tRNA$_{\mathrm{CUA}}$ is known to be robust to anticodon mutation.

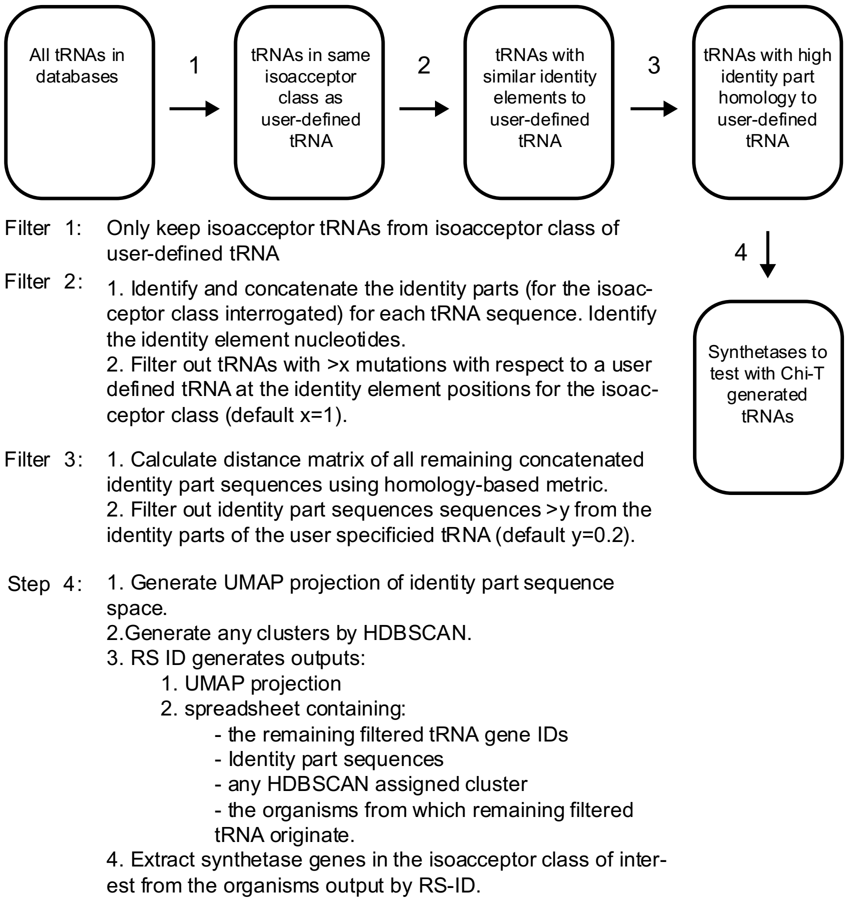

Supplementary Figure 11. Pseudocode of the RS-ID script.

RS-ID applies a series of filters to the tRNA database that enable the user to identify synthetase genes to test with the chimeric tRNAs generated by Chi-T. In the first step isoacceptors based on the tRNA sequence, it aims to identify tRNAs with similar ID-parts to the input tRNA. For tRNAs from isoacceptor classes with multiple ID parts RS-ID generates a concatenated reference of all individual identity parts, which is used as a reference. This reference is filtered for its distance in the specific identity base position in the reference tRNA as well as overall sequence homology to the concatenated reference. The script generates a list of potential tRNAs and corresponding genomes for which the contained synthetases can be selected for screening of Chi-T generated tRNA designs.

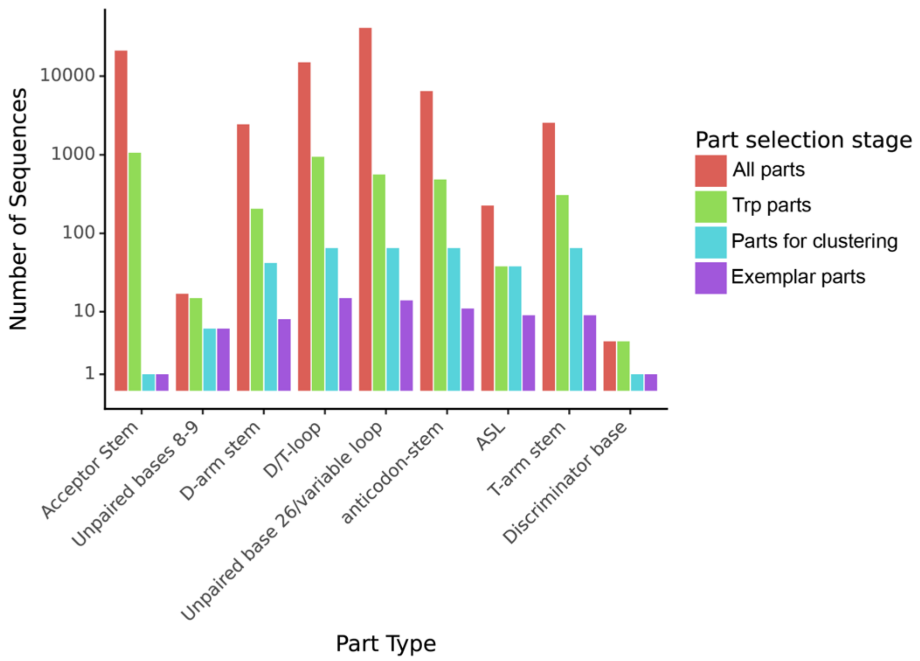

Supplementary Figure 12. The reduction in part number through part selection.

Bar graph showing the number of sequences in each part pool for each part type. The example shown is for tryptophanyl tRNAs with identity parts from *Eh*^{Trp}tRNA$_{CUA}$. All parts: the number of unique sequences in the cleaned tRNADB-CE data; Trp parts: the number of sequences from 'All parts' that are derived from tryptophanyl-tRNAs; Cluster parts: the number of sequences fed into the clustering algorithm after part identity scoring – note the acceptor stem and discriminator base part types reduce to one here, as they are identity parts, thus only one sequence is used and fixed throughout Chi-T; Exemplar parts: the number of sequences chosen as cluster exemplars through clustering (Affinity Propagation).

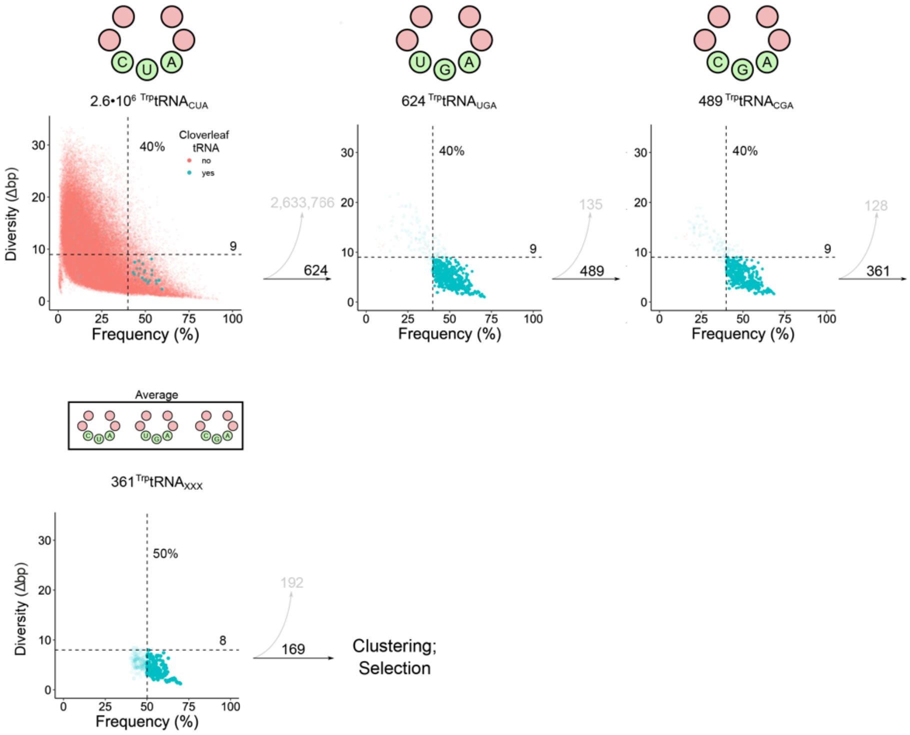

Supplementary Figure 13. Filtering chimeric $^{Trp}tRNA_{CUA}$ sequences with $Eh^{Trp}tRNA$ identity parts, to identify sequences that are robust to anticodon mutation.

The library of 2.6 • 10^6 chimeric $^{Trp}tRNA_{CUA}$ sequences (generated within Chi-T) is folded by RNAfold. The resulting MFE structure frequency (x), ensemble diversity (y) and cloverleaf structure (blue: cloverleaf, red: other) of these tRNAs is shown. Cloverleaf tRNAs with frequency > 40% and diversity < 9 Δbp (represented by solid circles) were selected within Chi-T. The anticodon sequence of the 642 selected tRNAs was altered from CUA to UGA, the resulting sequences were folded and selected with the same filters. The anticodon sequence of the 489 selected tRNAs was altered to CGA, and the resulting sequences were folded and then selected with the same filters. This process yielded 361 sequences. The frequency and diversity of the anticodon variants of these 361 sequences (with CUA, UGA, and CGA anticodons) was used to calculate the average frequency and diversity for each sequence across all three anticodons. The 169 tRNAs with an average frequency > 50%, and an average ensemble diversity < 8 Δbp were put forward for clustering.

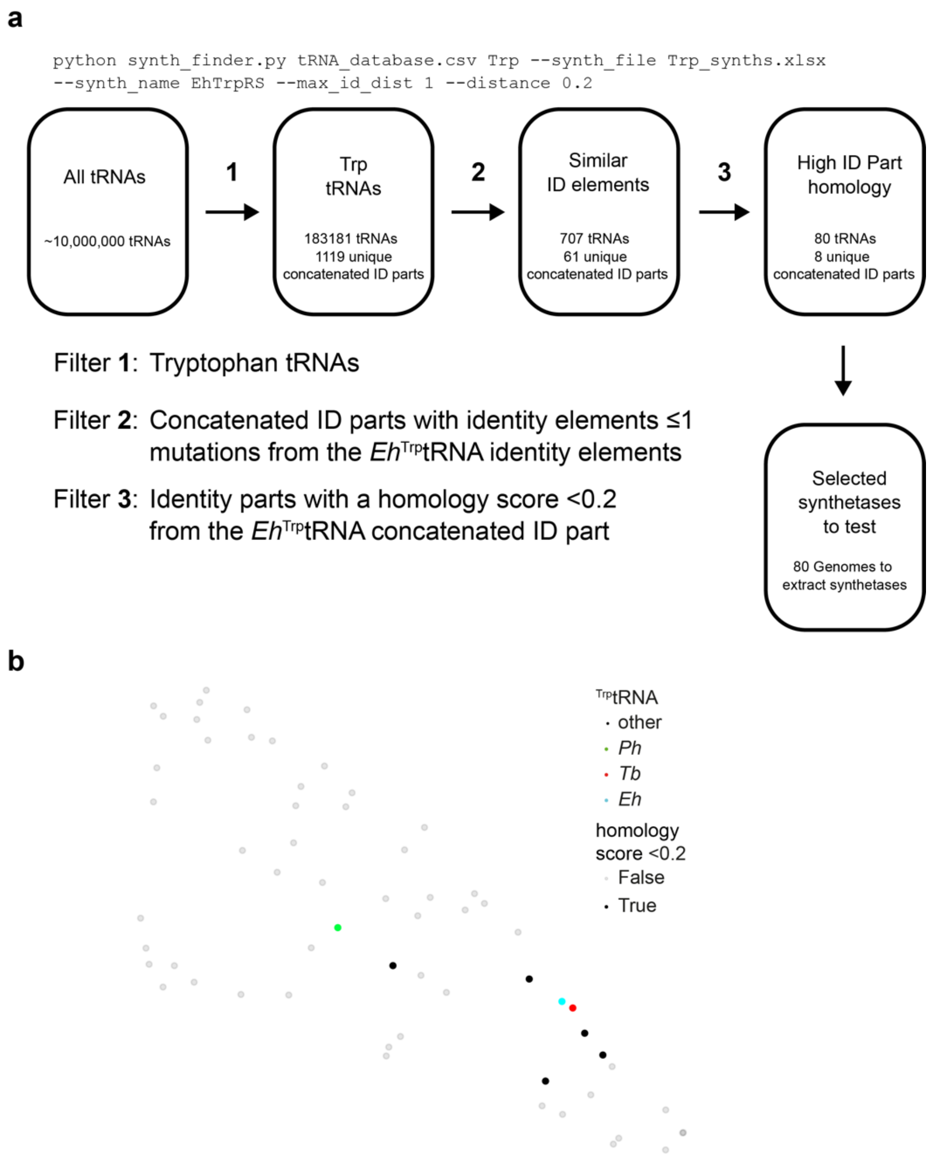

Supplementary Figure 14. RS-ID script for *Eh*^{Trp}tRNA identity parts.

a) RS-ID (**Methods**, **Supplementary Figure 11**) was run using the *Eh*^{Trp}tRNA sequence and the parameters given in the command line. **b**) UMAP projection of the unique concatenated identity part sequences of all tRNAs identified by RS-ID using *Eh*^{Trp}tRNA as an input. Unique identity part sequences of the selected tRNAs and input *Eh*^{Trp}tRNA are shown as coloured dots. All unique tRNA identity part sequences with a sequence similarity score <0.2 are solid black (or coloured) dots, all other sequences are translucent dots.

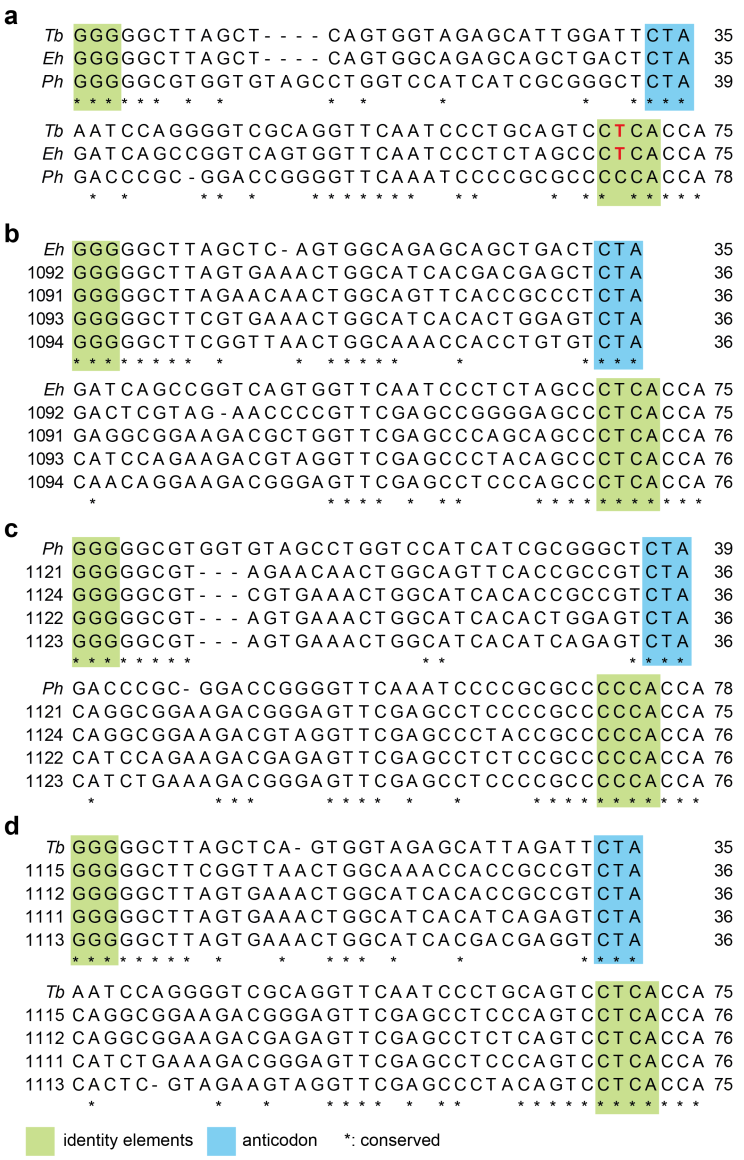

Supplementary Figure 15. Alignment of Chi-T input tRNAs and generated designs.

The sequences of the cognate tRNAs for the *Entamoeba histolytica* (*Eh*)TrpRS, *Trypanosoma brucei* (*Tb*)TrpRS and *Pyrococcus horikoshii* (*Ph*)TrpRS, and the sequences of the ^{Trp}tRNAs generated by Chi-T. CUA anticodon variants are shown. **a**) Sequence alignment of *Tb*^{Trp}tRNA$_{\mathrm{CUA}}$, *Eh*^{Trp}tRNA$_{\mathrm{CUA}}$ and *Ph*^{Trp}tRNA$_{\mathrm{CUA}}$. **b-d**) Sequence alignment of the Chi-T designed tRNA sequences for all three input tRNAs. Green shading: Identity elements for the tryptophanyl synthetases, Blue: CUA anticodon, red text: Difference between the *Tb*^{Trp}tRNA/*Eh*^{Trp}tRNA identity elements and the *Ph*^{Trp}tRNA identity elements, * indicates conserved bases.

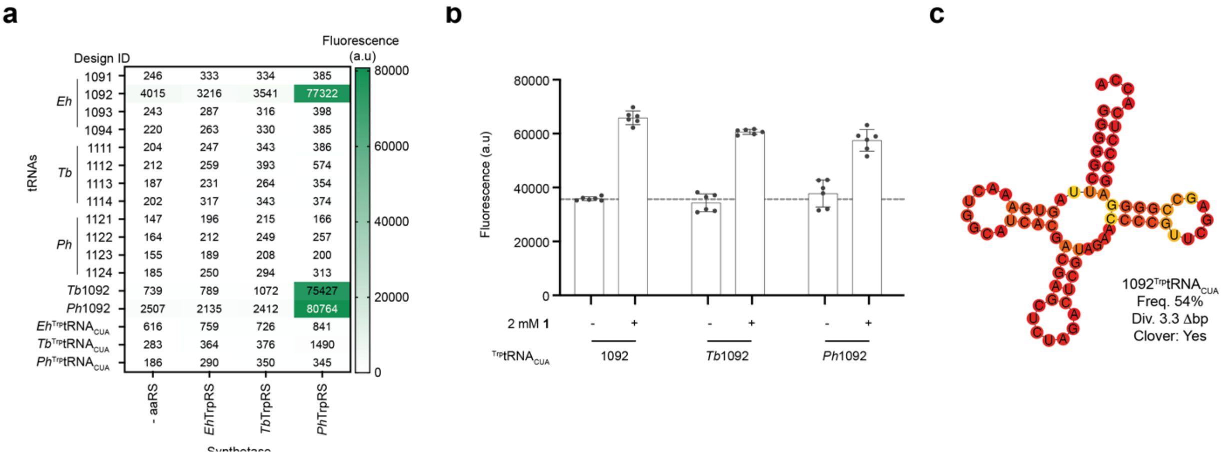

Supplementary Figure 16. Screening of Chi-T designed tRNAs, with identity parts from *Tb*^{Trp}tRNA, *Eh*^{Trp}tRNA and *Ph*^{Trp}tRNA, with synthetases identified by RS-ID.

The genes for Chi-T generated tRNA sequences were cloned into a vector (with or without a synthetase gene) and introduced into cells containing a *sfGFP150TAG*$_{His6}$ reporter. **a**) GFP fluorescence normalised by OD_{600} of cells containing the indicated tRNA and synthetase genes. Design *Tb*1092 and *Ph*1092 have the tryptophanyl identity part (acceptor stem) sequence of *Eh* replaced with the respective part sequence of *Tb*^{Trp}tRNA or *Ph*^{Trp}tRNA (the other tryptophanyl-identity part, the discriminator base, is the same for all three ^{Trp}tRNAs). Values for single measurements from the pool of transformed cells are shown. **b**) GFP fluorescence from cells bearing *sfGFP150TAG*$_{His6}$ of *Ph*TrpRSv1* and the indicated 1092^{Trp}tRNA$_{\mathrm{CUA}}$ in the absence or presence of 2 mM **1**. The dots represent the measurement from six independent replicates and the error bars show the standard deviation from the mean. The dashed line indicates the level of GFP fluorescence generated from *sfGFP150TAG*$_{His6}$ by the *Mm*PylRS/Mm^{Pyl}tRNA$_{\mathrm{CUA}}$ with 2 mM AllocK. (**c**) RNAfold prediction of 1092^{Trp}tRNA$_{\mathrm{CUA}}$. The base colour indicates the probability of the pairing status of each base as it appears in the MFE structure. The calculated MFE structures' frequency (%), diversity (Δbp) and cloverleaf classification (yes/no) are given.

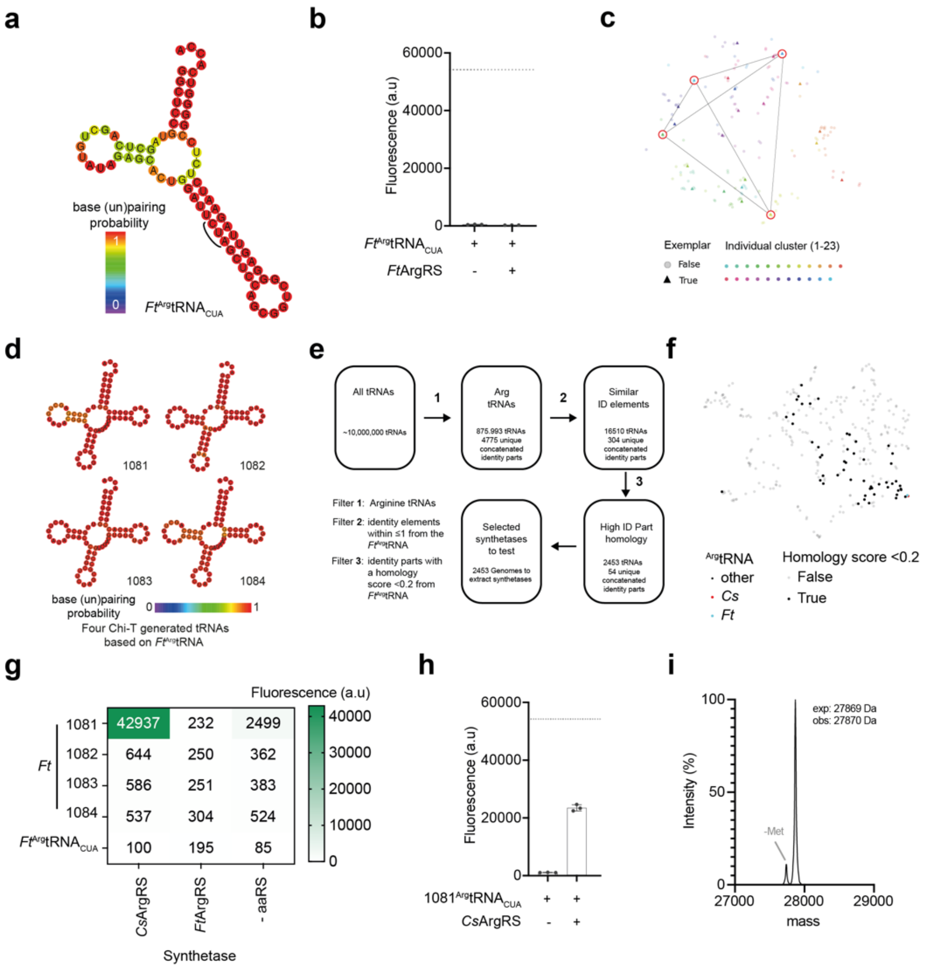

Supplementary Figure 17. Generation of 1081^{Arg}tRNA$_{CUA}$, an active orthogonal arginyl-tRNA, by Chi-T. And, the RS-ID led identification of a synthetase (*Cs*ArgRS) that functions with 1081^{Arg}tRNA$_{CUA}$ a) RNAfold-predicted MFE structure of the *Fluviicola taffensis* DSM 16823 arginyl-tRNA with its anticodon (black line) changed from UCG to CUA. Base colouring represents the predicted probability that a base has the pairing status predicted by the MFE structure. **b**) GFP fluorescence from *sfGFP150TAG$_{His6}$* measured in cells containing. *Ft*^{Arg}tRNA$_{CUA}$ with no synthetase (-) or *Fluviicola taffensis* arginyl-tRNA synthetase *(Ft*ArgRS*)*. The experiments were performed in three independent replicates. The individual data points are shown as dots, the bars represent mean values and the error bars indicate the s.d.. **c)** 167 tRNAs from Chi-T were clustered to identify the four most distant tRNA designs from 23 cluster exemplars, the group of four selected (red circles) has a

minimum of 16 mutations between any pair of tRNAs. **d**) The RNAfold-predicted MFE structures and sequences of the four selected arginyl-tRNAs. **e**) RS-ID run using Ft^{Arg}tRNA identity parts as input to identify potential cognate synthetases for the Chi-T generated chimeric tRNAs. Overall, 54 concatenated sets of identity elements passed all filters, these correspond to 2453 unique synthetase sequences. **f**) UMAP projection of the RS-ID result. We selected *Capnocytophaga sp. oral taxon 329 str. F0087* (*Cs*)ArgRS (red dot) to screen alongside the *Ft*ArgRS (blue dot); *Cs*ArgRS is active in *E. coli* with its native tRNA[1]. Other potential candidates within the homology score (<0.2) are solid black dots and other sequences are depicted as translucent dots. All unique concatenated identity part sequences in the figure are within one identity element mutation of $Ft^{Arg}tRNA_{CUA}$. **g**) GFP fluorescence, normalised by OD_{600} of cells, with the indicated synthetase and tRNA combinations. Values are for single measurements from cells transformed with the relevant plasmids. **h**) GFP fluorescence from cells bearing $sfGFP150TAG_{His6}$ and $1081^{Arg}tRNA_{CUA}$ in the absence and presence of *Cs*ArgRS. The dots represent the measurement from three independent replicates and the error bars show the standard deviation from the mean, the grey dotted line is the average activity of the *Mm*PylRS/$MmPyltRNA^{opt}_{CUA}$ in presence of 2 mM AllocK. **i**) Mass spectrometry of sfGFP purified from cells expressing $1081^{Arg}tRNA_{CUA}$ with *Cs*ArgRS and $sfGFP150TAG_{His6}$. Expected mass of *sf*GFP incorporating arginine at position 150 is 27869 Da the observed mass is 27870 Da.

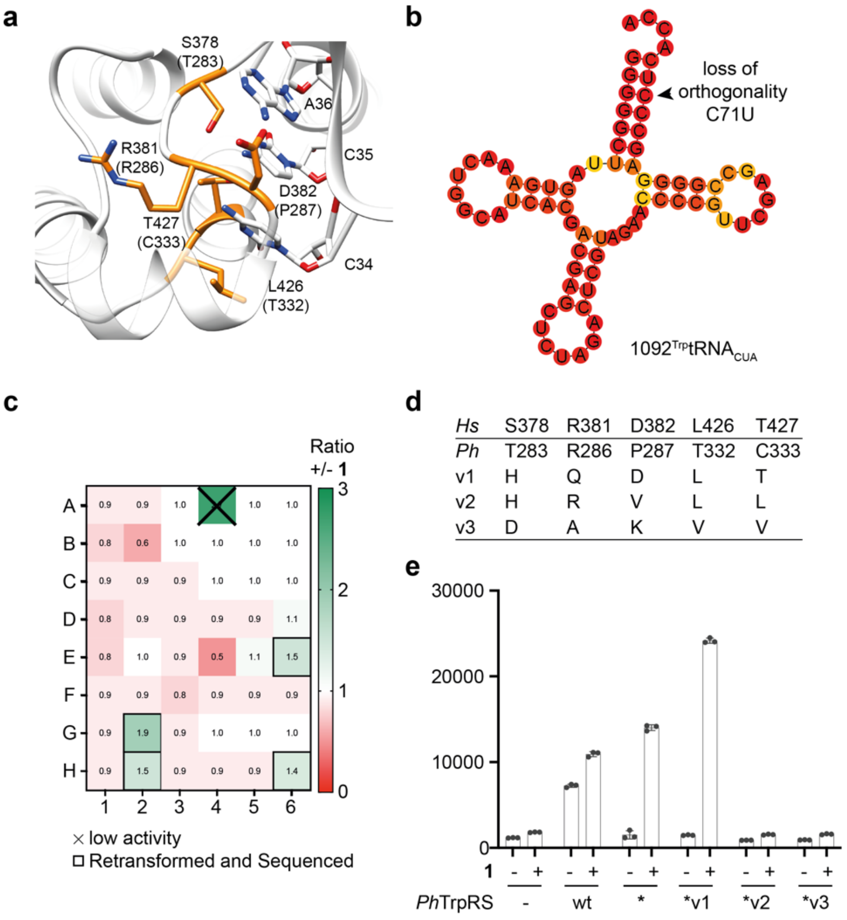

Supplementary Figure 18. Directed evolution of *Ph*TrpRS* anticodon recognition for 1092^{Trp}tRNA$_{CUA}$

a) Anticodon recognition in the *Homo sapiens* (Hs) tryptophanyl-tRNA synthetase bound to *Hs*^{Trp}tRNA$_{CCA}$ (PDB: 2AKE[2]). Anticodon bases (white) and residues targeted (orange) for mutagenesis in the anticodon recognition library are marked. The corresponding residues in *Ph*TrpRS* are shown in brackets. The ACR library was selected in the same manner as for *Ap*HisRS and *Cs*ProRS (see **Supplementary Fig. 6** and **7**). **b**) MFE structure of 1092^{Trp}tRNA$_{CUA}$. In the selection for *Ph*TrpRS* variants a large number of colonies were obtained which carried an escape mutation C71U, marked with an arrow. This mutation renders the tRNA non-orthogonal. **c**) Clones obtained from the selection were pre-screened for dependence on 5-OH tryptophan (**1**) before they were retransformed and sequenced. The heatmap shows the ratio of GFP fluorescence normalized by OD_{600} in presence and absence of

1. Crossed wells were discarded due to overall low activity, outlined wells were selected for retransformation and sequencing. **d**) Sequencing of transformants obtained from wells E6, G2, H2 and H6 revealed three *Ph*TrpRS* variants (v1-v3). **e**) GFP fluorescence, normalised by OD_{600}, of cells containing $1092^{Trp}tRNA_{CUA}$ and no synthetase (-), *Ph*TrpRS (wt), *Ph*TrpRS*, or its variants (v1 to v3) in the absence or presence of 2 mM **1**; cells contain *sfGFP3TAG*$_{His6}$. All measurements were performed in independent triplicates. The dots represent the individual measurements, the bars represent the mean values and the error bars show the standard deviation from the mean.

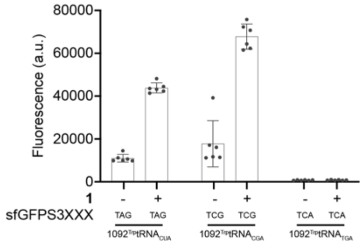

Supplementary Figure 19. Decoding activity of 1092^{Trp}tRNA anticodon variants in Syn61Δ3(ev5).

GFP fluorescence, normalised by OD_{600}, of Syn61Δ3(ev5) containing 1092^{Trp}tRNA$_{CUA}$ (or CGA, TGA anticodon variants, as indicated) with *Ph*TrpRSv1* in the absence or presence of 2 mM **1**. Cells contained *sfGFP3TAG-His6* (or the indicated variants in which the TCG or TCA codon replace the TAG codon). The *Ph*TrpRS*v1/ 1092^{Trp}tRNA$_{UGA}$ pair was inactive on TCA codons, consistent with two of the three anticodon bases in 1092^{Trp}tRNA$_{CUA}$ that are recognized by *Ph*TrpRS*v1 being mutated. Future work may show it is possible to use directed evolution to generate a PhTrpRS variant that is functional with 1092^{Trp}tRNA$_{UGA}$. Measurements of two separate experiments performed in triplicates, are shown. The dots represent the individual measurements, the bars represent the mean values and the error bars show the standard deviation from the mean.

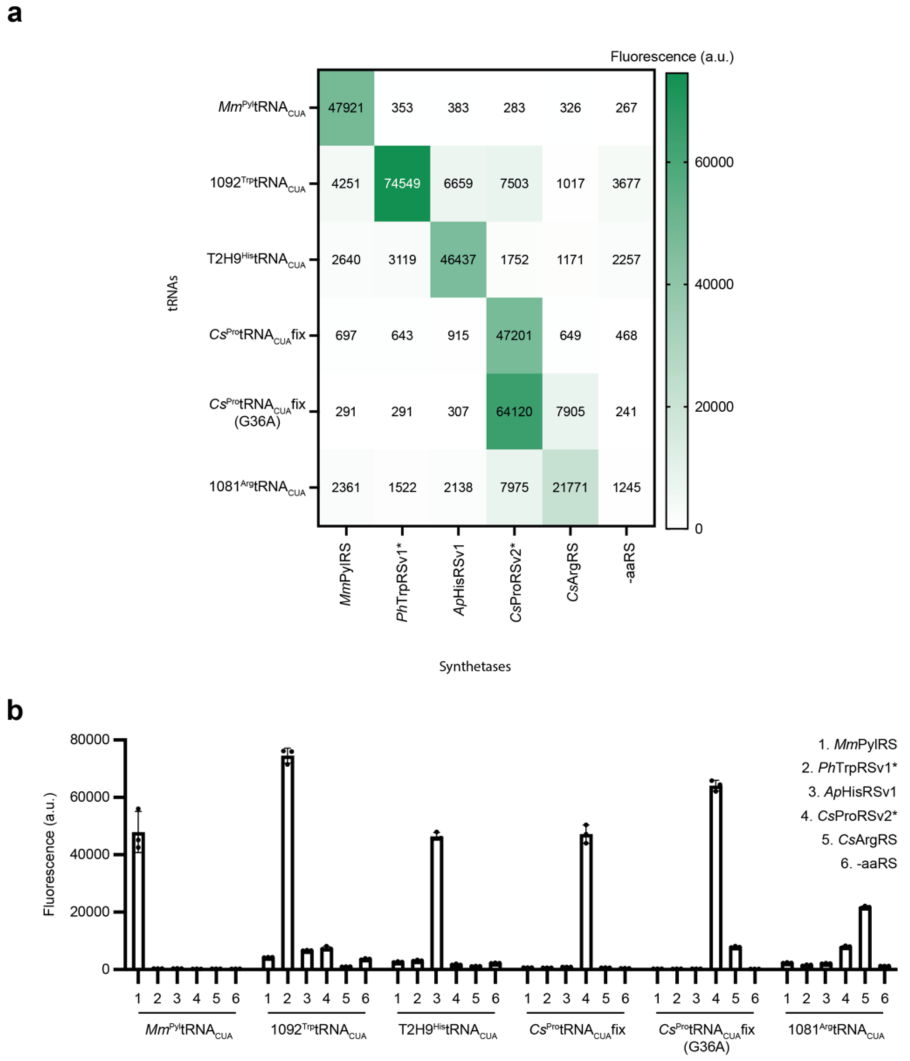

Supplementary Figure 20. Determining mutual orthogonality of the tRNA/-synthetases pairs reported in this work. a) GFP fluorescence for amber suppression of a *sfGFP150TAG*$_{His6}$ reporter gene measured from cells containing the indicated combinations of synthetases and tRNAs. Measurements with *Mm*PylRS and PhTrpRSv1* were made with 2 mM AllocK or 2 mM **1**, respectively. Each square shows the mean fluorescence intensity of three replicates in arbitrary units (a.u.), values were normalized by optical density (OD_{600}). **b**) Individual measurements (dots) and standard deviation of the mean for all combinations summarized in panel **a**. The experiment was performed in three independent replicates.

Supplementary Note 1. Native tRNA MFE folding and activity.

Approximately 80% of orthogonal-active tRNAs are predicted to fold into a cloverleaf-structure, while 41.6% of the primary sequences of orthogonal inactive tRNAs were predicted to fold into cloverleaf tRNAs; it is unclear if these tRNAs are inactive because the cognate synthetase in not stably expressed or because they are not recognized by their cognate synthetase in *E. coli*. Only 20% of undetectable tRNAs have primary sequences that are predicted to fold into a cloverleaf-structure; many of these sequences may be unfolded and degraded in cells.

Our data suggests that orthogonal and active tRNAs are enriched in primary RNA sequences that form predictable MFE cloverleaf structures (**Fig. 1b**); why might this be? The predicted MFE structures of tRNAs may not correspond to the cloverleaf structures they form in their natural host organism, as several host factors may enhance tRNA folding and structure [3,4].

However, when expressed outside of the native host (eg: in *E. coli*) the enzymes that modify the tRNAs in their native hosts are absent and the intrinsic ability of the tRNAs to fold becomes more important. So, while native and non-orthogonal tRNAs may benefit from tRNA modification or other cellular interactions to achieve a cloverleaf structure, orthogonal-active tRNAs may rely primarily on the intrinsic properties of their sequence to achieve cloverleaf folding in heterologous hosts. If this hypothesis holds true, MFE cloverleaf folding is a distinct feature of active and orthogonal tRNAs.

References

1 Cervettini, D. *et al.* Rapid discovery and evolution of orthogonal aminoacyl-tRNA synthetase–tRNA pairs. *Nature Biotechnology* **38**, 989-999 (2020). https://doi.org:10.1038/s41587-020-0479-2

2 Shen, N., Guo, L., Yang, B., Jin, Y. & Ding, J. Structure of human tryptophanyl-tRNA synthetase in complex with tRNATrp reveals the molecular basis of tRNA recognition and specificity. *Nucleic Acids Research* **34**, 3246-3258 (2006). https://doi.org:10.1093/nar/gkl441

3 Helm, M. *et al.* The presence of modified nucleotides is required for cloverleaf folding of a human mitochondrial tRNA. *Nucleic Acids Research* **26**, 1636-1643 (1998). https://doi.org:10.1093/nar/26.7.1636

4 Lorenz, C., Lünse, C. E. & Mörl, M. tRNA Modifications: Impact on Structure and Thermal Adaptation. *Biomolecules* **7**, 35 (2017).
